# The architecture of the SARS-CoV-2 RNA genome inside virion

**DOI:** 10.1038/s41467-021-22785-x

**Published:** 2021-06-24

**Authors:** Changchang Cao, Zhaokui Cai, Xia Xiao, Jian Rao, Juan Chen, Naijing Hu, Minnan Yang, Xiaorui Xing, Yongle Wang, Manman Li, Bing Zhou, Xiangxi Wang, Jianwei Wang, Yuanchao Xue

**Affiliations:** 1 Key Laboratory of RNA Biology, Institute of Biophysics, Chinese Academy of Sciences, Beijing, China; 2 University of Chinese Academy of Sciences, Beijing, China; 3 National Health Commission of the People’s Republic of China Key Laboratory of Systems Biology of Pathogens and Christophe Mérieux Laboratory, Institute of Pathogen Biology, Chinese Academy of Medical Sciences & Peking Union Medical College, Beijing, China; 4 CAS Key Laboratory of Infection and Immunity, Institute of Biophysics, Chinese Academy of Sciences, Beijing, China; 5 School of Life Sciences, Henan Normal University, Xinxiang, China; 6 State Key Laboratory of Stem Cell and Reproductive Biology, Institute of Zoology, Chinese Academy of Sciences, Beijing, China; 7 Institute for Stem Cell and Regeneration, Chinese Academy of Sciences, Beijing, China; 8 Key Laboratory of Respiratory Disease Pathogenomics, Chinese Academy of Medical Sciences and Peking Union Medical College, Beijing, China

**Keywords:** Sequencing, SARS-CoV-2, Virus structures

## Abstract

SARS-CoV-2 carries the largest single-stranded RNA genome and is the causal pathogen of the ongoing COVID-19 pandemic. How the SARS-CoV-2 RNA genome is folded in the virion remains unknown. To fill the knowledge gap and facilitate structure-based drug development, we develop a virion RNA in situ conformation sequencing technology, named vRIC-seq, for probing viral RNA genome structure unbiasedly. Using vRIC-seq data, we reconstruct the tertiary structure of the SARS-CoV-2 genome and reveal a surprisingly “unentangled globule” conformation. We uncover many long-range duplexes and higher-order junctions, both of which are under purifying selections and contribute to the sequential package of the SARS-CoV-2 genome. Unexpectedly, the D614G and the other two accompanying mutations may remodel duplexes into more stable forms. Lastly, the structure-guided design of potent small interfering RNAs can obliterate the SARS-CoV-2 in Vero cells. Overall, our work provides a framework for studying the genome structure, function, and dynamics of emerging deadly RNA viruses.

## Abstract (web summary)

Secondary structures and long-range RNA interactions of the SARS-CoV-2 genome have been investigated by various sequencing methods. Here the authors use an RNA-RNA hybrid sequencing method to predict the secondary and tertiary structure of the SRAS-CoV-2 RNA genome in the virion.

## Introduction

Severe acute respiratory syndrome coronavirus 2 (SARS-CoV-2) is the causal pathogen of coronavirus disease 2019 (COVID-19)^1–3^. As a single-stranded and positive-sense RNA virus, SARS-CoV-2, together with SARS-CoV and Middle East respiratory syndrome coronavirus (MERS-CoV), all belong to the *Coronaviridae* family^4^. SARS-CoV-2 carries one of the largest RNA genomes (~30 kilobases, kb) among all RNA virus families and encodes about 29 proteins^1,3,5–7^. Since its outbreak in late December 2019, SARS-CoV-2 has infected tens of millions of people and caused over one million deaths worldwide (https://covid19.who.int/). Although global efforts and resources have been redirected to fight against SARS-CoV-2, there are no effective antiviral medicines available yet. Considering the RNA nature of SARS-CoV-2, RNA-based therapeutics such as small interference RNAs (siRNAs) or antisense oligos (ASOs) are emerging as potent agents to cleave the viral RNA genome in infected host cells. Because RNA structure can significantly influence the efficacy of siRNAs and ASOs^8,9^, deciphering the 3D structure of SARS-CoV-2 becomes an urgent need prior to RNA-based drug development.

RNA structures are widely recognized as critical modulators in regulating transcription, translation, and replications of coronavirus and other RNA viruses^10–17^. At this frontier, many efforts have been devoted to study the structure of SARS-CoV-2. Even though Cryo-electron microscopy and 3D electron tomography are powerful in delineating the global architectures of SARS-CoV-2, the entire RNA genome inside virions remains unrevealed^18–20^. Besides these physical approaches, several chemical-based high-throughput sequencing methods, such as in vivo click selective 2′-hydroxyl acylation and profiling experiment (icSHAPE) and dimethyl sulfate mutational profiling with sequencing (DMS-MaPseq), have been recently applied to probe single-stranded regions of SARS-CoV-2 in infected cells or in vitro^21–24^. The mapped single-stranded information could be further used as restraints to predict the base-paired regions within 500 nt^25^. Yet the current secondary structural model of SARS-CoV-2 might be incomplete since it missed information of long-range duplexes, which are prevalent and vital for completing the life cycles of positive-strand RNA viruses^26,27^. As a significant advance, a psoralen-based method called cross-linking of matched RNAs and deep sequencing (COMRADES) recently identified many long-range RNA duplexes of SARS-CoV-2 inside cells, further highlighting the importance of RNA duplexes in maintaining SARS-CoV-2 fitness^28,29^. Significantly lagging behind those in-cell structural studies, how the 30 kb genome of SARS-CoV-2 is organized and arranged in virions remains unclear.

We recently developed an RNA in situ conformation sequencing technology, named RIC-seq, for unbiased mapping of RNA-RNA spatial interactions in living cells^30^. RIC-seq utilizes a pCp-biotin to label proximally interacting chimeric RNAs and high-throughput sequencing to retrieve their spatial proximity information. We demonstrated that RIC-seq could successfully detect short- and long-range duplexes, multiple-way junctions, and loop-loop contacts without base pairing potentials. These merits make RIC-seq more competent to decipher the 3D structure of the SARS-CoV-2 RNA genome. But SARS-CoV-2 virions are typically 80 nm in diameter and can’t be pelleted down as human cells by standard centrifugation^1^. This feature makes virions not compliant with our current RIC-seq protocol that includes extensive washing and standard centrifugation at every enzymatic step^30^. To overcome the major challenges, we designed a way to trap SARS-CoV-2 virions on Concanavalin A (ConA) beads that bind specifically to glycoproteins present on the surface of virions. By optimizing virion capture, crosslinking, and enzymatic conditions, we further developed a virion RNA in situ conformation sequencing technology, named vRIC-seq, for global mapping of viral RNA genome structures in intact virions.

In this study, we successfully applied the vRIC-seq technology to probe the SARS-CoV-2 RNA genome structure in intact virions. We reconstructed a model of the surprisingly compact yet unentangled tertiary structure of the SARS-CoV-2 RNA genome. At the secondary structure level, we found that the overall structure of SARS-CoV-2 is more compacted in virion than that in the host cell, and long-range RNA duplexes are prevalently present. The covariant analysis revealed that many long-range RNA-RNA interactions are under purifying selection and might contribute to the 3D package of the SARS-CoV-2 RNA genome. Finally, we uncovered several highly accessible single-stranded regions in SARS-CoV-2 for efficient viral RNA cleavage in Vero cells by using siRNAs.

## Results

### Overview of vRIC-seq technology

**Fig. 1:**
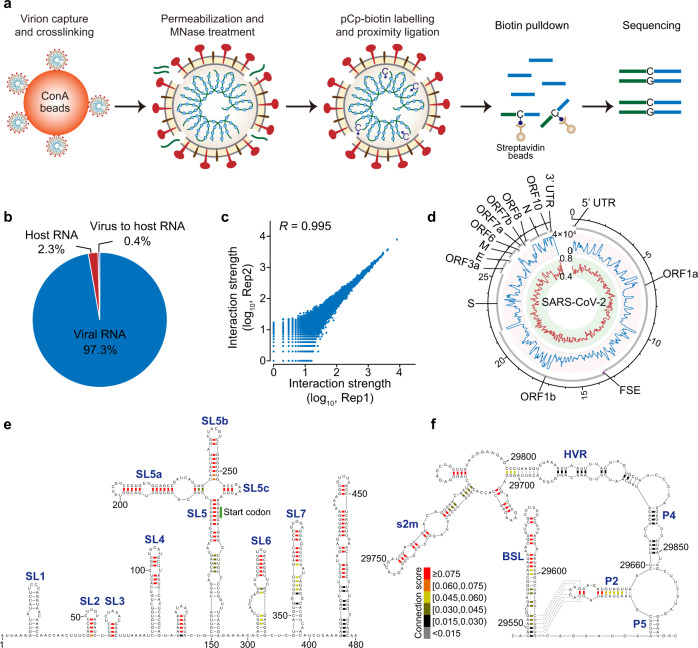
**Overview and evaluation of vRIC-seq technology**. **a** Scheme of vRIC-seq technology. Concanavalin A (ConA) beads were used to capture the virion for diverse enzyme treatments in subsequent steps. **b** The proportions of chimeric reads mapped to SARS-CoV-2. **c** Scatter plots showing the correlation between two biological replicates for the number of chimeric reads (interaction strength). *R*, Pearson correlation coefficient. **d** Circos plot showing the distribution of chimeric reads along the SARS-CoV-2 genome. The inner red circle stands for the fractions of adenine or uracil within 100 nt windows, and the outer blue circle shows the coverage of chimeric reads. FSE frame-shifting element. **e**, **f** vRIC-seq confirmed known coronavirus RNA structures in the 5′ UTR (1–480 nt, **e**) and 3′ UTR (29,546–29,870 nt, **f**) of the SARS-CoV-2 RNA genome. Connection scores shown in different colors were used for assessing the base-pairing probability. The dashed lines illustrated the pseudoknot.

Like the other coronaviruses, the envelope of SARS-CoV-2 contains two major glycoproteins spike (S) and membrane (M)^31,32^. According to this feature, we used magnetic beads coated with ConA, a plant lectin that can specifically bind glycoproteins, to capture the SARS-CoV-2 virions prepared from the supernatants of infected Vero cells (see “Methods”). After capturing the virions on beads, formaldehyde was further applied to crosslink the nucleocapsid (N) protein-mediated proximal RNA-RNA interactions, as well as ConA and the surface glycoproteins (Fig. 1a). Next, the virions were permeabilized and treated with micrococcal nuclease (MNase) to digest single- and double-stranded RNAs protruded from the nearby protein complexes. Subsequently, all the proximal RNAs were 3′ end-labeled with pCp-biotin and ligated together by T4 RNA ligase. Lastly, the resulting chimeric RNAs marked with pCp at the juncture were enriched and converted into libraries for paired-end sequencing (about 260 bp, Fig. 1a and Supplementary Fig. 1a).

We obtained ~24.6 million unique reads for each replicate and ~3.4 million chimeric reads resulting from different RNA fragments. Using the RIC-seq data analysis workflow established earlier^30^, we found that 97.3% of the chimeric reads could be aligned to the SARS-CoV-2 genome, and 2.3% were mapped to host RNA (Fig. 1b). The trace amounts of host RNA reads might be derived from detached Vero cells at the virion collection stage. Notably, ~0.4% of the chimeric reads were virus-to-host RNA interactions (Fig. 1b), reflecting the random ligation rates between virions and Vero cells or representing interactions between viral and host RNAs that happened inside the detached Vero cells. Besides, the virus-to-host interactions also raised the possibility that some host RNAs might be packaged into the virion, just like tRNA in the HIV virions for its replication^33^. However, such a possibility was excluded because we observed a random virus-to-host RNA interaction pattern across all the green monkey chromosomes (Supplementary Fig. 1b).

The pCp-biotin labeling and selection were successful because ~85% of the additional nucleotides at chimeric junctions were cytosine (Supplementary Fig. 1c). vRIC-seq is highly reproducible on chimeric reads coverage (*R* = 0.999) and interaction strength (*R* = 0.995) of pairwise sites (Fig. 1c and Supplementary Fig. 1d). Moreover, we noted that 99.7% of the SARS-CoV-2 genome was covered at least 2500× by chimeric reads (Supplementary Fig. 1e), and the remaining 0.3% covered by <2500× was mainly located at the stem-loop 1 of 5′ UTR and the poly (A) region at the 3′ terminal. Next, we aligned the pairwise interacting RNA fragments to the SARS-CoV-2 genome, and such analysis revealed many regions that are preferably cut by MNase and subsequently labeled with pCp-biotin (Fig. 1d). We also noticed that the first 3500 nucleotides (nt) of ORF1b had a relative lower vRIC-seq coverage, and nucleotide content didn’t contribute to this difference (Fig. 1d).

### Recapitulate known structures of the SARS-CoV-2 genome

We first determined to validate the viral RNA spatial interactions revealed by vRIC-seq. For this purpose, we deduced several conserved RNA duplexes in coronavirus using vRIC-seq data and compared it with recently proposed SARS-CoV-2 secondary structure models^13,21–24,34–36^. As expected, vRIC-seq faithfully recapitulated all of the stem-loops in the 5′ UTR (1–395 nt) of SARS-CoV-2 except stem-loop 1 (SL1), which is ~40 nt in length and can’t be purified by AMPure XP beads (Fig. 1e). In addition, the 3′ UTR of SARS-CoV-2 contains several conserved structural elements known to functionally impact viral RNA synthesis and translation, including the stem-loop II-like motif (s2m), hyper-variable region (HVR), and mutually exclusive bulged stem-loop (BSL) or pseudoknot (PK)^36^. We found that vRIC-seq successfully captured the canonical s2m and HVR structures (Fig. 1f). However, in contrast to the previous theoretical model^36^, our data preferentially supported a double hairpin conformation for BSL and P2 stem, rather than the PK conformation (Fig. 1f). These results demonstrate that vRIC-seq can faithfully probe RNA spatial interactions in the virion, and support that this proximity information can be used for structure modeling.

### Topological organization of the SARS-CoV-2 genome

**Fig. 2:**
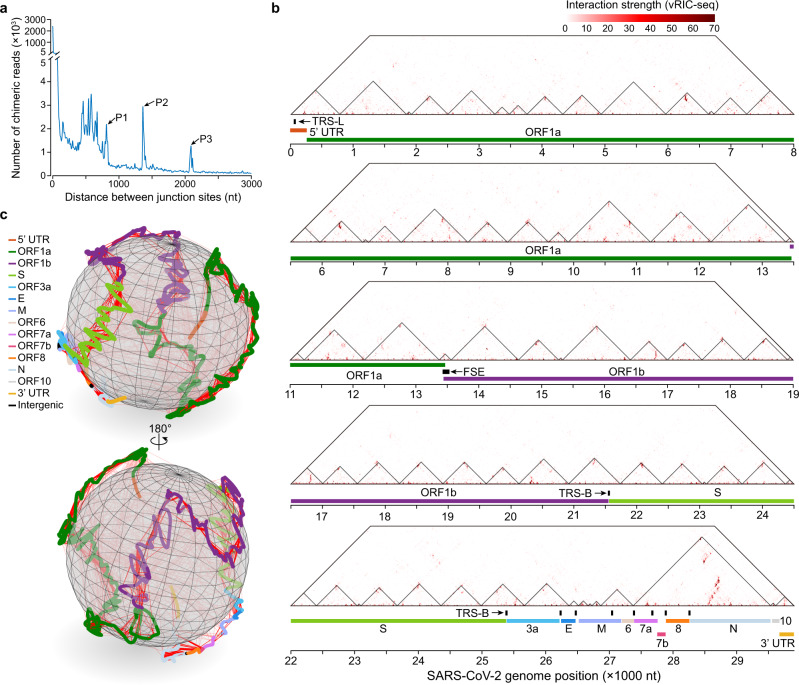
**Global view of SARS-CoV-2 genome organization**. **a** Spanning distance of pairwise interacting RNAs. P1, P2, and P3 mark three peaks corresponding to chimeric interactions spanning 810, 1360, and 2090 nucleotides. **b** RNA interaction map of the SARS-CoV-2 genome. The black triangles represent RNA topological domains. The frame-shifting element (FSE), the transcription-regulatory sequence in the leader (TRS-L), and the body (TRS-B) are marked as black lines. **c** The global configuration of the SARS-CoV-2 RNA genome in virions, modeled by the miniMDS software. The 30 kb RNA genome of SARS-CoV-2 is presented as a rope, and each coding region and UTR are marked with different colors. The vRIC-seq detected RNA contact frequencies were used for the modeling. The solid red lines represent chimeric signals that support the local interactions, whereas the dashed red lines depict long-range interactions. The same picture rotated in 180 degrees is shown at the bottom.

After validating the vRIC-seq data, we next investigated the features of SARS-CoV-2 genome organization in the virion. To this end, we first calculated the spanning distance of pairwise interacting RNA fragments. Approximately 90.6% of the pairwise interactions happened within 100 nt, whereas ~6.3% of the interactions spanned more than 600 nt, and three sharp peaks with 810, 1360, and 2090 nt separately, were observed (Fig. 2a). Of note, those long-distance interactions were not caused by the discontinuous transcription of SARS-CoV-2 during negative-strand synthesis^6^, because we observed a clear enrichment of pCp at the chimeric junctions (see red lines, Supplementary Fig. 2a, b).

Following our previous approach, we divided the SARS-CoV-2 genome into 10-nt windows and constructed an RNA interaction map (Fig. 2b). Using the map, we identified 254 clustered interactions positioned perpendicular to the diagonal, suggesting the widespread occurrence of local duplexes in the SARS-CoV-2 genome (Fig. 2b). Surprisingly, we also noticed 77 long-range interactions that were sequentially distributed and covered almost the entire genome (Fig. 2b). Similar to the organization of human primary transcripts^30^, we observed 49 RNA topological domains with a median length of 630 nt in the SARS-CoV-2 genome (Fig. 2b, Supplementary Fig. 2c, and Supplementary Data 1). The largest domain is located before the 3′ UTR and contains sequences encoding ORF7a, ORF7b, ORF8, and the N protein (Fig. 2b).

Like RIC-seq technology^30^, vRIC-seq can also capture base-paired RNA duplexes and protein-mediated indirect RNA contacts. To examine the base-pairing probabilities for the observed long-range interactions within 2.5 kb, we calculated the minimum free energy (MFE) for pairwise interacting fragments that spanned over 400 nt. We observed significantly lower MFE values than randomly shuffled sequences with the same nucleotide content (*P* = 1.62e−10, Supplementary Fig. 2d), indicating that those long-range RNA–RNA interactions may be directly base-paired. Notably, we also uncovered 62 pairwise interacting RNA fragments that could span over 2.5 kb (Supplementary Fig. 2e and Supplementary Data 2). 69.4% (43/62) of those long-range interactions were supported by more COMRADES^29^ reads than the randomly selected genomic-span-matched controls (Supplementary Fig. 2f). However, those long-range duplexes seemed not the preferred conformation in virions because the individual RNA fragments showed 12-fold stronger interactions with their local partners than distal partners (Supplementary Fig. 2g). For example, long-range interactions between 1180–1190 nt and 29,343–29,354 nt, both showed stronger local interactions (Supplementary Fig. 2h–j). These data suggest that some alternative topology of the SARS-CoV-2 genome might be present in the virion.

### 3D globule configuration of the SARS-CoV-2 genome in virions

Previous microscopy studies revealed a spherical shape with a mean diameter of ~80 nm for CoV and SARS-CoV-2 viral particles^1,37,38^. To explore how the 30 kb RNA genome of SARS-CoV-2 is folded in the tiny virion, we utilized the contact frequencies data of different RNA fragments as constraints to model the global conformation of the SARS-CoV-2 genome. Pursuing this, we adopted a widely used miniMDS (multidimensional scaling) approach to infer the 3D structure of SARS-CoV-2^39^. Our modeling revealed a 3D globule conformation of the RNA genome (Fig. 2c and Supplementary Video 1). Notably, the thread-like genome was unentangled, appearing to have a mildly helical conformation. Moreover, different segments of the viral genome seemed to occupy separate territories (see different colors, Fig. 2c), forming an organization apparently similar to known structures of mammalian genomes^40^. Of note, this “globule” and knot-free configuration might reflect the arrangement of the N protein, which has been shown to bind the CoV genome and interacted with M protein via its C-terminal domain in the interior of the lipid membrane of virions^18^.

We next tried to use the in silico folded or in-cell structure model to simulate the 3D configuration of the SARS-CoV-2 genome^28,36^. We found that the in silico model contains only 3196 base-pairs within 120 nt and covered ~28% of the SARS-CoV-2 genome^36^. The lack of long-range RNA-RNA interactions prevents us from simulating the 3D configuration of the SARS-CoV-2 genome using the in silico model. For viral RNA duplexes revealed by COMRADES^28^, 76.9% showed multiple alternative RNA-RNA interactions in Vero cells (Supplementary Fig. 2k). By contrast, vRIC-seq revealed that 88.9% of the SARS-CoV-2 RNA duplexes in virions showed one dominant interaction (Supplementary Fig. 2k). Moreover, the Shannon entropy (higher Shannon entropy represents more alternative interactions) for each 10-nt genomic window was 6.08 in host cells by COMRADES and 2.35 in virion by vRIC-seq (Supplementary Fig. 2l). The higher structural flexibility and heterogeneity of SARS-CoV-2 genomic RNAs inside cells blocked us for further simulation. These results collectively highlight the values of vRIC-seq data in modeling viral RNA genome structure inside the virions.

### Secondary structure model of the SARS-CoV-2 genome

Based on the 3D RNA interaction map, we further developed an adaptive strategy to model the secondary structure of the entire SARS-CoV-2 genome. Briefly, we first predicted local duplexes, which were positioned in our SARS-CoV-2 RNA interaction map as perpendicular signals to the diagonal, and then used these duplexes as constraints to predict long-range duplexes (Supplementary Fig. 3a). To evaluate the performance of this strategy, we first predicted the secondary structure of 28S rRNA using our previously published RIC-seq data in HeLa cells^30^. The structural model achieved a sensitivity of 83.0% and a positive predictive value (PPV) of 78.3%, and both criteria were significantly higher than structures merely based on the minimal free energy values provided by several computational tools (Supplementary Fig. 3b). Importantly, our algorithm showed higher PPV and sensitivity if RIC-seq detected duplexes spanning over 600 nt were counted (Supplementary Fig. 3c). Together, these data demonstrate the accuracy of our adaptive strategy in deducing RNA structures.

**Fig. 3:**
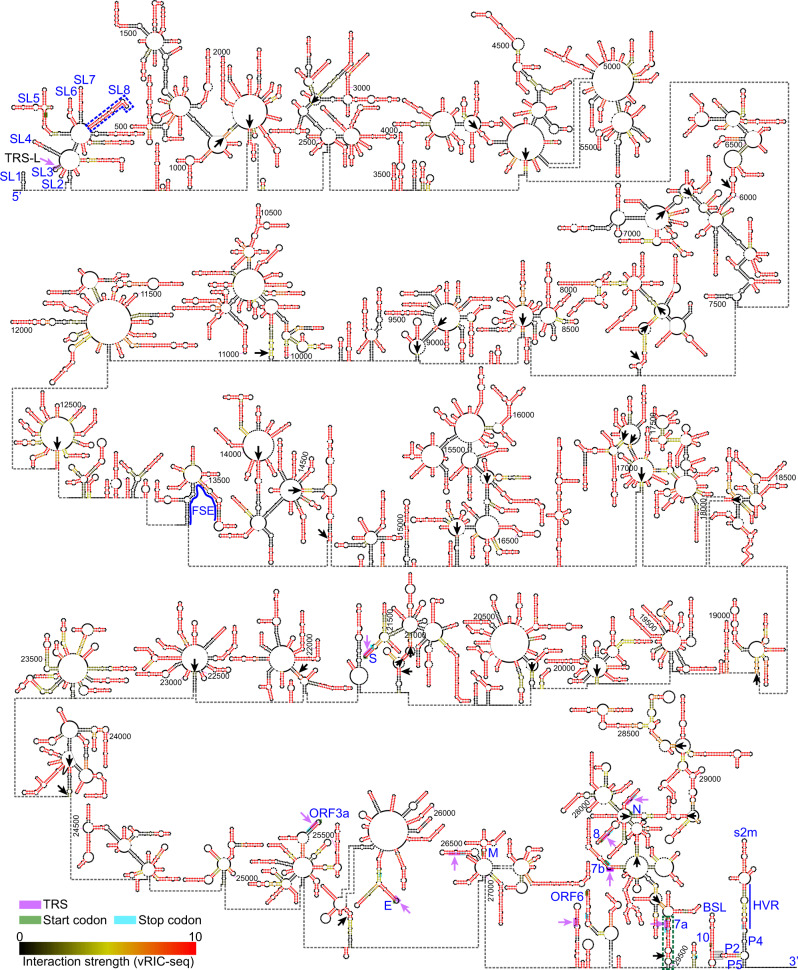
**The secondary structure of the SARS-CoV-2 genome**. The known structural elements in the 5′ UTR, the frame-shifting element (FSE), and the 3′ UTR are labeled or marked in blue. The pairwise interaction strength was quantified and shown in different colors. Black arrows highlight highly confident long-range duplexes measured by vRIC-seq signals. Green and cyan boxes mark the start and stop codons, respectively. Purple boxes and arrows outline the core sequence (CS) of each transcription-regulatory sequence (TRS).

Having validated the prediction algorithm, we next applied it to reconstruct the secondary structure of the whole SARS-CoV-2 genome (Fig. 3). Our structural model was highly favored by the vRIC-seq data, as base-paired regions showed stronger interaction strength than size-matched unpaired control sequences (*P* < 2.2e−16, Supplementary Fig. 3d). In our structural model, the median and mean distances between two paired regions in SARS-CoV-2 were 28 and 111 nt, respectively. The maximal spanning distance (2145 nt) was observed for a duplex formed between two fragments: 27,357–27,396 nt and 29,465–29,502 nt (green dash line boxed region, Fig. 3). We found that about 63.8% (19,064 nt) of the SARS-CoV-2 genome were base-paired, and our model precisely recapitulated the stem-loop structures (SL1-SL7) in the 5′ UTR, as well as 3′ UTR structures including the s2m and the HVR (Fig. 3). Notably, our vRIC-seq data strongly supported an extended duplex SL8, rather than the two previously proposed separate duplexes which were theoretically predicted using the coding sequence of nsp1 (410–470 nt)^36^ (see blue dash line marked region, Fig. 3). Besides identifying many duplexes, our structural model unexpectedly revealed 167 multi-way junctions, including 57 three-way junctions and three 12-way junctions; these junctions seemed to organize the SARS-CoV-2 RNA genome into many petaloid structures (Fig. 3 and Supplementary Fig. 3e).

Next, we examined whether there were any correlations between structural elements and viral RNA abundance. During negative-strand RNA synthesis, SARS-CoV-2 produces nine subgenomic RNAs (sgRNAs) via the template-switching activity of RNA-dependent RNA polymerase (RdRP) between the 5′ leader sequence and the transcription-regulatory sequence in the body (TRS-B)^6^. Interestingly, the TRS-B of ORF7a was located in a long-range duplex spanning over 2000 nt, while the other eight TRS-B were located in local stem-loops (purple boxed regions, Fig. 3 and Supplementary Fig. 3f). Moreover, we found that the number of single-stranded nucleotides within TRS-B was negatively correlated to the abundance of the corresponding sgRNA (*R* = −0.52, Supplementary Fig. 3g). By contrast, the number of single-stranded nucleotides in regions adjacent to the TRS-B showed positive correlations with sgRNA levels (Supplementary Fig. 3h). It will be interesting to examine how these structural features determine viral RNA transcriptions in the future.

### The in-cell and in-virion structural dynamics

**Fig. 4:**
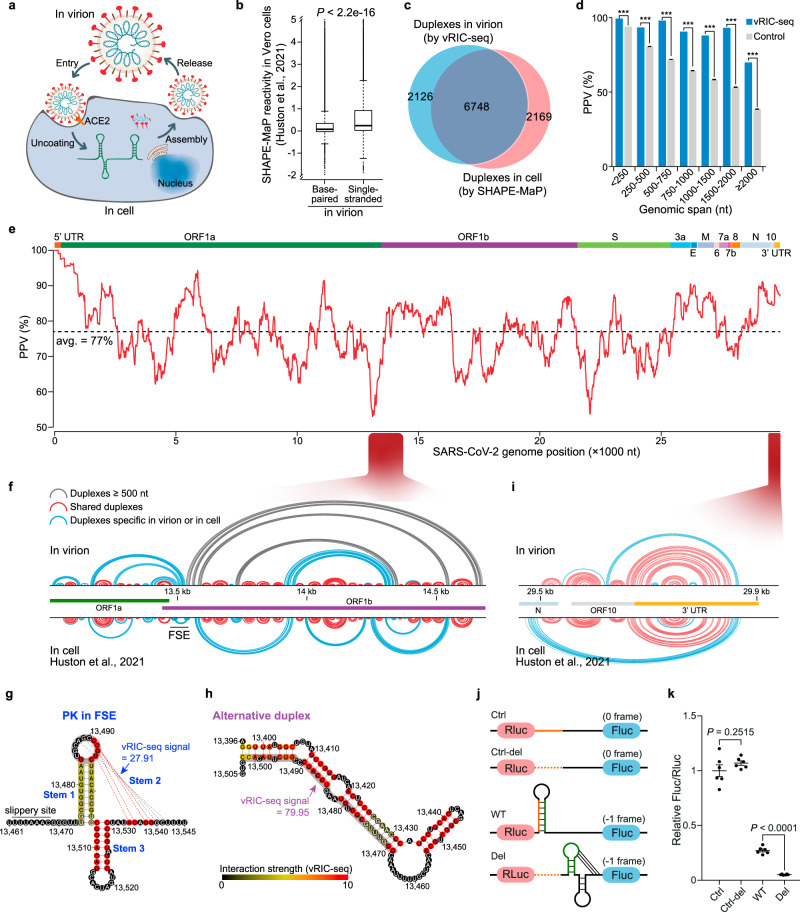
**Comparison of SARS-CoV-2’s structure in virions and host cells**. **a** Schematic diagram showing the life cycle of SARS-CoV-2. **b** Single-stranded nucleotides (*n* = 8269) identified by vRIC-seq in virions have higher SHAPE-MaP reactivities than base-paired nucleotides (*n* = 19055) in cells. *P*-value was determined by two-tailed, unpaired Student’s *t*-test. The center line of the box plot represents the median, the box borders represent the first (Q1) and third (Q3) quartiles, and the whiskers are the most extreme data points within 1.5× the interquartile range (from Q1 to Q3). **c** Venn diagram showing the overlap between duplexes revealed in virions and cells. **d** The percentage of in-virion interactions supported by the COMRADES chimeric reads is decreased along with the spanning distance. The control datasets are randomly selected pairwise loci (repeated 1000 times, *n* = 1000). Data are mean ± s.e.m., ***P < 0.001, one-sided permutation test. **e** The positive predictive value (PPV) of the in-virion duplexes compared to the in-cell predicted duplexes along the SARS-CoV-2 genome in sliding 1 kb windows. The dashed line indicates the average percentage. **f** In-virion (top) and in-cell (bottom) duplexes in the FSE surrounded region. Arc lines colored in gray indicate base pairs spanning more than 500 nt, arc lines in red indicate base pairs shared by duplexes in virions and cells, while arc lines in blue indicate base pairs specific in virions or cells. **g** Diagram the pseudoknot structure of FSE. The dashed lines represent base pairs in Stem 2. Different colors in bases stand for the strength of vRIC signals. The scale is shown at the right bottom. An alternative duplex can form at the gray shadowed regions. **h** vRIC-seq data preferably supports an elongated duplex in the FSE region. **i** In-virion (top) and in-cell (bottom) duplexes in 3′ UTR surrounded region. **j** Dual-Luciferase Reporters for functional characterization of the alternative duplex. Before the slippery site, a 33 nt sequence (orange) can form a duplex with the Stem 1 (green) of FSE-PK. In-frame is denoted as “0 frame”, while “−1 frame” stands for programmed −1 ribosomal frameshift (−1 PRF). The dashed orange lines stand for the deleted 33 nt sequence. Ctrl control, WT wild type, Del deletion, Rluc renilla luciferase, Fluc firefly luciferase. **k** The alternative duplex can stimulate −1 PRF activity. Data are mean ± s.e.m., *P*-value was determined by a two-tailed, unpaired Student’s *t*-test (*n* = 6 biologically independent experiments). Source data are provided as a Source Data file.

SARS-CoV-2 can hijack host cells by engaging its spike proteins with the host receptor angiotensin-converting enzyme 2 (ACE2)^7^. After entering into the host cell, the SARS-CoV-2 genome is released from the densely coated N proteins for translating nonstructural proteins to replicate its genome, as well as to produce structural proteins to assemble new virions (Fig. 4a). Whether there is any genome structure remodeling before and/or after the infection is still unclear. By analyzing the in-cell SHAPE-MaP (selective 2′-hydroxyl acylation analyzed by primer extension and mutational profiling) data^24^, we found that the single-stranded regions revealed by vRIC-seq tended to have stronger SHAPE signals than base-paired regions (*P* < 2.2e−16, Fig. 4b). Unexpectedly, 76% of the duplexes identified by vRIC-seq in virion might also present in host cells by comparing with the SHAPE-derived structural model (Fig. 4c). In addition, over 88% of the in-virion duplexes that happened within 2 kb were also present in host cells, evidenced by COMRADES identified chimeric reads (Fig. 4d). However, the pairwise interactions over 2 kb showed evident remodeling in the host cells (Fig. 4d).

Notably, the duplexes in the 5′ UTR regions barely changed in virions and the host cells (Fig. 4e). One of the most dynamic regions was 12.6–13.6 kb, which covered the frame-shifting element (FSE) and resided at the boundary of ORF1a and ORF1b. Although the in silico modeling^36^, phylogenetic analysis^12,41^, cryo-EM study^42^, and in-cell structure^24^ all supported the presence of this PK conformation, our in-virion data strongly suggested an alternative duplex preferably formed between the Stem 1 of FSE-PK and an upstream 33 nt sequence (Fig. 4f–h and Supplementary Fig. 4a). Consistent with our observation, another study indirectly predicted this alternative duplex conformation in infected cells by using DMS-MaPseq to reveal single-stranded information^23^. Both conformations might be dynamically present, but we observed 2.9-fold more vRIC-seq signals to support the alternative duplex than the PK (79.95 vs. 27.91, Fig. 4g, h) in virions. We also examined another well-known and unstable PK in the 3′ UTR of betacoronavirus^13,43^. Our vRIC-seq data barely support the presence of the 3′ UTR PK, and both the in-virion and in-cell structures predict an extended BSL rather than a PK (Fig. 3, Fig. 4i, and Supplementary Fig. 4b–d).

We next determined to test whether the newly identified alternative duplex could modulate the activity of FSE-PK-mediated programmed −1 ribosomal frameshift (−1 PRF). We used a Dual-Luciferase Reporter assay to measure the −1 PRF activity in 293T cells^41^. The FSE sequence was first inserted between renilla and firefly luciferase to make a WT vector, denoted as −1 frame (Fig. 4j). Subsequently, we deleted the 33 nt upstream sequences to create a “Del” vector to see how it influence PK-mediated −1 PRF activity. To measure the −1 PRF efficiency, we also made two in-frame control plasmids by mutating the slippery site or deleting the 33 nt sequence in the in-frame backbone. These two plasmids were denoted as “Ctrl” and “Ctrl-del”, respectively (Fig. 4j). Next, we transfected those four plasmids individually into the 293T cells and allowed luciferase gene expression 24 h. Compared with the Del plasmid, we found that including this 33 nt sequence could stimulate −1 PRF activity by 5.7-fold (4.8% vs 27.2%), suggesting the critical requirement of this alternative duplex in maintaining high-efficiency −1 PRF (Fig. 4k). Together, these results demonstrate the accuracy of our SARS-CoV-2 secondary structure model in virions and highlight the potentially impactful structural dynamics during infection.

### Co-variants in duplexed regions of SARS-CoV-2

RNA duplexes usually are under selection pressure to maintain the viral genome’s conformation to facilitate its replication^17^. To uncover duplexes under purifying selection, we first conducted an analysis to examine the co-variant base pairs in 429 non-redundant coronavirus genomes. This comparison revealed 406 sequence covariations (Supplementary Data 3). Eight co-variant base pairs were located at SL5-6 in the 5′ UTR, and 15 co-variant base pairs were at the HVR in the 3′ UTR (Supplementary Fig. 5a, b). Next, we classified all the co-variant events into four categories: (1) detected in both in-virion and in-cell duplexes^24^ (Class I); (2) detected in both in-virion and in silico predicted duplexes^36^ (Class II); (3) detected in the in-virion, in-cell, and in silico duplexes (Class III); (4) detected only in the in-virion duplexes (Class IV). We found that ~32% of the co-variants were only observed in our in-virion duplexes (Supplementary Fig. 5c). Those in-virion-specific duplexes could span longer distances than other kinds of co-variants (Supplementary Fig. 5d). Importantly, we detected 13 duplexes that spanned more than 600 nt and showed focused co-variations among multiple strains (Supplementary Fig. 5e–g). These results highlight the importance of long-range duplexes revealed by vRIC-seq in maintaining viral RNA structure and potential fitness.

**Fig. 5:**
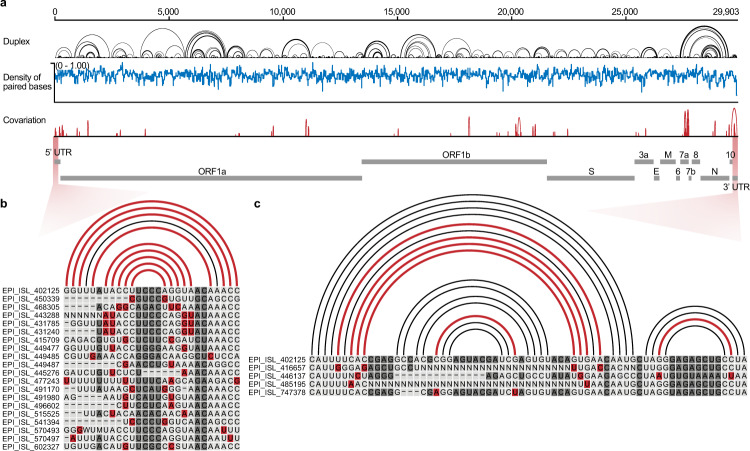
**Co-variation among duplexes in different SARS-CoV-2 strains**. **a** Profile of structural elements and variants in the SARS-CoV-2 genome. The black arc lines stand for base pairs, the blue line indicates the density of base-paired nucleotides, and the red arc lines denote co-variant base pairs among different SARS-CoV-2 strains. **b**, **c** Co-variant base pairs in the 5′ UTR (**b**) and 3′ UTR (**c**), respectively. Arc lines and nucleotides colored in red indicate co-variant base pairs. EPI_ISL_402125 is the reference SARS-CoV-2 sequence.

Next, we investigated the co-variant base pairs among 200,621 SARS-CoV-2 strains. This analysis identified 98 co-variant events across the whole SARS-CoV-2 genome (see red arc lines, Fig. 5a and Supplementary Data 4), of which nine co-variants were located in SL1 and six in the s2m (see red shadowed regions in 5′ UTR and 3′ UTR, respectively. Fig. 5b, c). Moreover, we found 11 co-variant events in a three-way junction located in ORF7a (Supplementary Fig. 5h). To the 62 long-range interactions spanning over 2.5 kb, we observed that eight of them possessed at least one co-variant event (Supplementary Data 2 and 4). This evolutionary covariation analysis indicates that maintaining those structural elements might be necessary for viral fitness. Future investigations that experimentally determine the function of each covariation could help elucidate the practical impact(s) of these structurally fascinating elements.

### D614G and accompanying mutations on structure remodeling

**Fig. 6:**
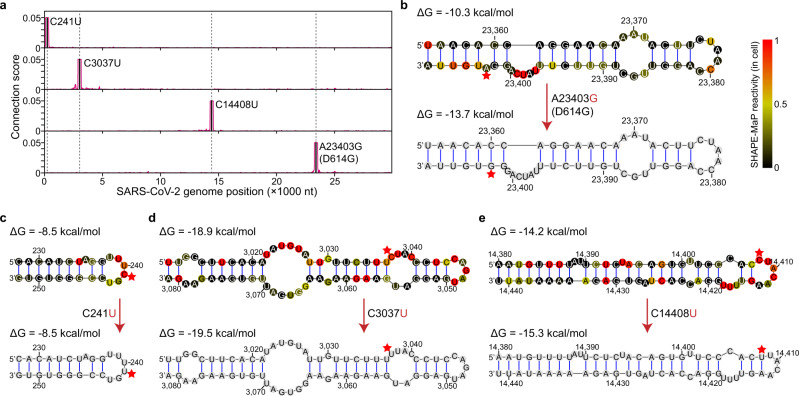
**D614G and the accompanying mutations on structure remodeling**. **a** Line plot showing the point mutation resided regions tend to form local interactions. The C241U, C3037U, C14408U, and A23403G (D614G) mutants are marked as solid lines. **b** The A-to-G transition (D614G mutant) at 23,403 nt remodels two bulge structures into a single six-nucleotide bulge. Red stars mark the mutated nucleotides. Δ*G*, free energy. **c**–**e** The D614G accompanying mutations have no influence on duplexes except for the C14408U transition (**e**).

SARS-CoV-2 genome is frequently mutated during evolution due to lacking proofreading activity of RdRP. Some mutations are beneficial to SARS-CoV-2 and emerge as dominant strains in the global pandemic, such as D614G and three accompanying mutations^44^. We found that these four mutations were located in local duplexed regions, and no interactions could be observed between them (Fig. 6a). The most prevalent D614G mutant caused by an A-to-G nucleotide transition at position 23,403, was located in the single-nucleotide bulge of a stem-loop (Fig. 6b). Interestingly, we found that the A23403G mutation fine-tuned the two local bulge structures into a thermodynamically more favorable six-nucleotide bulge structure (−10.3 kcal/mol vs. −13.7 kcal/mol) (Fig. 6b). Agreeing well with our model, the three uracil (U) in the bulge also showed strong SHAPE-MaP signals in the SARS-CoV-2 infected cells^24^. The D614G mutation had been demonstrated to increase the infectivity and stability of virions^45^. Besides the amino acid substitution in the spike protein, the remodeled bulge structures might also contribute to these effects.

In addition, the D614G accompanying mutations at 241, 3037, and 14,408 nt all resided in the single-stranded internal or apical loops (Fig. 6c–e). These loops could also be partially supported by the SHAPE-MaP signals revealed in the host cells^24^. However, the C14408U mutation in RdRP (P323L) might introduce a novel structure with a smaller apical loop (14,380–14,441 nt) (Fig. 6e). It might be worthwhile to study further how these mutations are introduced during infection and how they enable viral fitness advantage. In addition, we systematically analyzed all the observed mutations in different SARS-CoV-2 strains. We found that the base-paired nucleotides were less mutated and had slightly lower entropy scores. This phenomenon was also confirmed by analyzing in silico folded and in-cell structure models^24,36^ (Supplementary Fig. 6). Together, these results highlight the functional importance of maintaining duplexes during evolution.

### Structure-guided cleavage of SARS-CoV-2 RNA

**Fig. 7:**
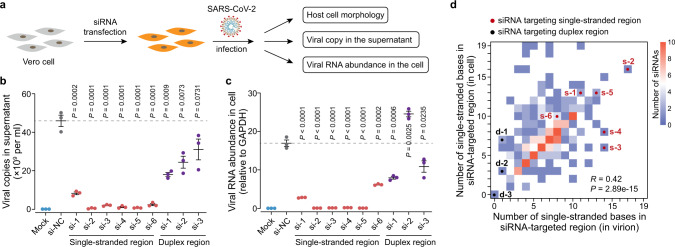
**Structure-guided design of potent siRNAs as a cleavage agent to restrict SARS-CoV-2 infection**. **a** Diagram of strategy against SARS-CoV-2 infection in Vero cells. **b** The SARS-CoV-2 copies in the supernatant were reduced to background level upon transfection with siRNAs targeting single-stranded regions (si-1 to si-6). Mock, uninfected cells; si-NC, non-targeting siRNA (control). **c** qPCR showing the abundance of viral RNA in infected Vero cells. Data in (**b**) and (**c**) are mean ± s.e.m.; *n* = 3 biological replicates, two-tailed, unpaired Student’s *t*-test. Source data are provided as a Source Data file. **d** Number of single-stranded bases within the siRNA target regions identified in cells and virions. The color intensity denotes the number of siRNAs. The six siRNAs targeting single-stranded regions and three siRNAs targeting duplex regions are labeled as s-1 to s-6 and d-1 to d-3, respectively. *R*, Pearson correlation coefficient. *P*-value was determined by two-sided correlation test.

RNA cleavage mediated by small interfering RNA (siRNA), antisense oligonucleotides, and RNA-targeting CRISPR, has emerged as a therapeutic modality to restrain viral gene expression and inhibit viruses in host cells^46^. However, it bears emphasis that target RNA cleavage efficiency is strongly affected by duplex structures^8,9^. As the in-virion structure revealed by vRIC-seq may be similar to the SARS-CoV-2 RNA genome configuration when virions just enter the host cells or at the final assembly stage (Fig. 4a), therefore designing siRNAs based on the in-virion structure may enable potent prevention of SARS-CoV-2 infection. In order to screen for cleavage-vulnerable regions of the SARS-CoV-2 RNA genome to develop anti-viral drugs, we systematically selected 130 single-stranded regions that (i) have at least 10 consecutive nucleotides and (ii) contain strong conservation among the different SARS-CoV-2 strains we examined (Supplementary Data 5). Next, we synthesized six siRNAs specifically targeting single-stranded regions and three siRNAs targeting duplex regions to cleave the SARS-CoV-2 RNA genome, and conducted infectivity assays using Vero cells (Fig. 7a).

qPCR analysis of cells at 24 h post viral exposure showed that, compared to the non-targeting siRNA control (denoted as NC), four out of the six tested siRNAs targeting single-stranded regions could completely prevent SARS-CoV-2 amplification in Vero cells (see si-2, si-3, si-4, and si-5; Fig. 7b); accordingly, we observed no viral infection on cells treated with these four siRNAs (Fig. 7c). Agreeing well with the qPCR results, we found that the Vero cells treated with these four siRNAs looked healthier than those with the non-targeting siRNA control (Supplementary Fig. 7a). In sharp contrast, none of the siRNAs targeting duplexes could completely prevent infection or viral amplification (Fig. 7b, c). As each of the four effective siRNAs target sequences common to more than 90% of the 200,621 known SARS-CoV-2 strains, combining two or more of these siRNAs is expected to result in cleavage for nearly all known SARS-CoV-2 strains.

Importantly, we found that the structure of siRNA target regions revealed in virion is largely consistent with the structures uncovered in host cells (Fig. 7d). Furthermore, the siRNA targeting region designed by vRIC-seq showed more single-stranded base numbers than those from targeting regions selected by in silico methods (Supplementary Fig. 7b). This data highlight the SARS-CoV-2 RNA structure’s values in guiding the design of potent siRNAs. Together, these results validated the accuracy of vRIC-seq deduced structures and showcased its practical utility for supporting drug development to combat SARS-CoV-2 during the ongoing COVID-19 pandemic.

## Discussion

The emerging deadly RNA viruses have caused severe global epidemics and pandemics. RNA viruses usually form intricate and dynamic structures to orchestrate their translation, replication, and packaging^13,47^. Seeking to understand the in situ structure of viral RNA genome in virions, we developed vRIC-seq technology to map proximal contacts between different RNA fragments of the SARS-CoV-2. This global proximity information enabled us to construct a high-confidence RNA interaction map and build a 3D structure model of the SARS-CoV-2 genome, which is organized into an unentangled, globule, and seemingly spiral overall architecture. We also uncovered many short- and long-range duplexes, PKs, and multiway junctions in SARS-CoV-2 RNA. Unexpectedly, these long-range duplexes could further isolate the SARS-CoV-2 genome into RNA topological domains. Recently, many host RNA-binding sites of the SARS-CoV-2 genome have been revealed by the COMRADES method^28^. We found that these sites seem less accessible in virions than in cells, indicating that the viral RNA genome may need to undergo extensive structure remodeling for interacting with the host RNA molecules.

We also developed an adaptive algorithm to reconstruct a complete SARS-CoV-2 secondary structure model by integrating the 3D pairwise interactions of vRIC-seq data. This approach is significantly different from other widely used strategies that indirectly predict viral RNA duplex structures based on the nucleotide flexibility information^14,15,17,48,49^. Considering the duplex length restraint during prediction, the previously modeled structures of SARS-CoV-2 in the host cells might be incomplete because many functionally relevant long-range duplexes spanning over 600 nt were omitted^21–24^. It is worth mentioning that the in-virion structure seems relatively compact than in-cell structures revealed by SHAPE-MaP and other methods^21,23,24^. Several reasons may account for such a difference: (1) The ribosome machinery may extensively unfold the long-range and local duplexes of SARS-CoV-2 genomic RNA during translation in host cells; (2) the transcription of SARS-CoV-2 genomic and sgRNAs may also actively unwind many structural elements in infected cells; (3) many SARS-CoV-2 associated RNA helicases may extensively remodel viral RNA structures to accommodate its life cycles^50,51^; (4) RNA-binding proteins in host cells may bind and coat SARS-CoV-2 genomic RNA, thus preventing it from forming more compact configuration in host cells. The relatively compact state of the SARS-CoV-2 genome in virions may explain why the FSE PK is not the preferred structure. Functional validations demonstrate that our newly identified alternative duplex is required for high-efficiency −1 PRF activity. Although detailed mechanisms still need to be explored, our results suggest that PK’s structural dynamics may be necessary for frameshifting in the host cells.

The formation and maintenance of RNA duplexes are known to strongly influence viral fitness^52^. Thus, understanding single- and double-stranded regions in the viral genome is informative for the efficient development of RNA-targeted drugs to fight SARS-CoV-2 infections. The structural model we proposed here lays the foundation for developing and deploying highly potent siRNAs, single guide RNAs, and ASOs. Notably, a recent SARS-CoV-2 structure probed by SHAPE-MaP and DMS-MaPseq also identified 80 segments showing low structural variability and lack of base pairing. By comparing with our structural model, we found that 26 of the reported segments are overlapped with 23% (30/130) of the single-stranded regions defined by vRIC-seq (Supplementary Data 5). In addition, the three most potent siRNAs (si-3, si-4, and si-5) targeted single-stranded regions are not within the defined 80 segments. Moreover, the ineffective and duplexed region-specific siRNA (si-2) is assigned as a single-stranded segment in the paper. These comparisons further highlight the value of our in-virion structural model in guiding the design of RNA-based drugs.

Evolutionary sequence covariation is a strong indicator of the RNA duplex. vRIC-seq technology enabled us to identify more co-variants than in silico and in-cell predicted structure models. Our secondary structure model contains 51 long-range duplexes spanning more than 600 nt; 25.5% of them possessed at least one co-variant event during SARS-CoV-2 evolution (Supplementary Data 4), highlighting the functional importance of maintaining these structural elements for viral fitness. Our model also provides novel insights on how some pathogenic SARS-CoV-2 variants, such as D614G might enable a viral fitness advantage at the RNA structure level. However, it should be noted that the current structural model did not include binding information for the nucleocapsid (N) protein, which densely packages the SARS-CoV-2 genome into the ribonucleoprotein core in the interior of the virion^53^. Therefore, identification of the N protein’s binding sites along the genome may further narrow down the targeting regions to develop agents for efficient RNA cleavage.

In summary, we developed a high-throughput approach for probing the in situ genome structure of any RNA viruses theoretically. We further used SARS-CoV-2 as a model to illustrate the power of vRIC-seq in delineating its general architecture and sophisticated long-range RNA-RNA interactions, such as duplexes and multi-way junctions. The vRIC-seq approach is also easily adaptable to probe viral RNA structurome and interactome in host cells in the future. More importantly, we could also apply the vRIC-seq technology in profiling RNA spatial interactions with animal cells and plant cells. The inclusion of the capture step by ConA beads may significantly reduce the cell numbers used in our original RIC-seq protocol, thus enable RNA spatial interaction mapping with a small number of starting cells.

## Methods

### Experimental model and subject details

Vero cells (ATCC, CCL-81) were cultured in DMEM (Thermo Fisher, C11965500BT) containing 1% penicillin/streptomycin (Life Technologies, 15140) and 10% fetal bovine serum. All the live SARS-CoV-2 viruses-related experiments were carried out in the enhanced biosafety level 3 (P3+) facilities, which are authorized by the National Health Commission of the People’s Republic of China. The SARS-CoV-2 strain used in this study, IPBCAMS-YL01/2020, was isolated from a clinical sample by the team of Institute of Pathogen Biology, Chinese Academy of Medical Sciences & Peking Union Medical College. The virus was passaged three times in Vero cells before use. Infectious titers of SARS-CoV-2 were determined by plaque assay in Vero cells. SARS-CoV-2 virions were prepared as previously described and inactivated by β-propiolactone before bringing to the general laboratory for vRIC-seq analysis^54^.

### Virion capture and crosslinking

We used BioMag®Plus Concanavalin A beads (Polysciences Inc, 86057) to capture the SARS-CoV-2 virions via spike glycoprotein. To this end, 150 μl of ConA beads were first washed twice with binding buffer (20 mM HEPS-KOH pH 7.9, 10 mM KCl, 1 mM CaCl_2_, 1 mM MnCl_2_). The supernatant was discarded and the beads were resuspended in 400 μl of binding buffer. Subsequently, the 100 μl of virions were added to the tube and incubated for 10 min at room temperature (RT). After washing three times with binding buffer and once with PBST buffer (1× PBS, 0.1% Tween 20), the beads were resuspended in 1 ml of PBST buffer. To fix nucleocapsid (N) protein-mediated RNA–RNA interactions, 27 μl of 37% formaldehyde was applied and incubated for 10 min at RT with rotation at 20 rpm. The crosslinking was stopped by adding glycine to a final concentration of 0.125 M and incubated at RT for 5 min.

### Permeabilization and MNase treatment

The ConA beads and captured virions were resuspended in 1 ml of permeabilization buffer (10 mM Tris-HCl pH 7.5, 10 mM NaCl, 0.5% NP-40, 0.3% Triton X-100, 0.1% Tween 20, 1× protease inhibitors (Sigma Aldrich, P8340), 2 U/ml SUPERase In RNase inhibitor (Thermo Fisher Scientific, AM2694)), and rotated at 4 °C for 15 min at 20 rpm. After washing three times with 1× PNK buffer (50 mM Tris-HCl pH 7.4, 10 mM MgCl_2_, 0.1% Tween 20), the beads were further treated with 200 μl of 1 × MN mixture (50 mM Tris-HCl pH 8.0, 5 mM CaCl_2_, 0.03 U/μl micrococcal nuclease (Thermo Fisher, EN0181)) for 10 min at 37 °C. The MNase treatment was stopped by washing twice with 1× PNK + EGTA buffer (50 mM Tris-HCl pH 7.4, 20 mM EGTA, 0.1% Tween 20) and twice with 1× PNK buffer.

### pCp-biotin labeling and proximity ligation

The beads were gently resuspended in 100 μl of 1 × FastAP buffer containing 10 U of FastAP alkaline phosphatase (Thermo Fisher Scientific, EF0651) and incubated at 37 °C for 15 min. To stop the reaction, the beads were sequentially washed twice in 1× PNK + EGTA buffer, twice in 1× high-salt wash buffer (5× PBS, 0.1% Tween 20) (Pipette 6–8 times, this step should be brief), and three times in 1× PNK buffer. To deposit pCp-biotin, beads were resuspended in 100 μl of ligation mixture containing 10 μl of 10× RNA ligase reaction buffer, 6 μl of 40 U/μl RNase inhibitor, 4 μl of 1 mM pCp-biotin (Thermo Fisher Scientific, 20160), 100 U of T4 RNA ligase, 20 μl of nuclease-free water and 50 μl of 30% PEG 20000, and incubated overnight at 16 °C. After washing three times with 1× PNK buffer, the beads were resuspended in a PNK mixture (10 μl of 10× Imidazole buffer (500 mM imidazole-HCl pH 6.4, 100 mM MgCl_2_), 6 μl of 10 mM ATP, 4 μl of T4 polynucleotide kinase (Thermo Fisher Scientific, EK0032), 10 μl of 0.1 M DTT, 70 μl of nuclease-free water) and incubated at 37 °C for 45 min. Of note, T4 PNK possesses maximal kinase and 3′ phosphatase activity simultaneously in Imidazole buffer with pH 6.4^55^. Subsequently, the beads were washed twice with 1× PNK + EGTA buffer and twice with 1× PNK buffer. For in situ proximity ligation, the ligation mixture containing 20 μl of 10× RNA ligase reaction buffer, 8 μl of 40 U/μl RNA inhibitor (Thermo Fisher Scientific, EO0381), 10 μl of 10 U/μl T4 RNA ligase (Thermo Fisher Scientific, EL0021), 20 μl of 1 mg/ml BSA, and 142 μl of nuclease-free water was used to gently resuspend the beads and incubated overnight at 16 °C.

### RNA purification and library construction

On the next day, the ligation was stopped by washing three times with 1× PNK buffer. To extract viral RNAs, the beads were resuspended and incubated with 200 μl of proteinase K buffer (10 mM Tris-HCl pH 7.5, 10 mM EDTA, 0.5% SDS) and 50 μl of proteinase K (Takara, 9034) at 37 °C for 60 min and 56 °C for 15 min. The Eppendorf tube was placed on a magnet stand for 1 min, and the supernatant was transferred to a new tube. The viral RNA was extracted from the supernatant with 750 μl of TRIzol LS (Thermo Fisher, 10296028) and 220 μl of chloroform according to the manufacturer’s instruction. After centrifugation at 16,000 g for 15 min at 4 °C, the supernatant was transferred to a 1.5 ml Eppendorf tube and mixed with 500 μl of isopropanol and 1 μl of glycoblue (15 mg/ml, Thermo Fisher, AM9515). The RNA was precipitated overnight at −20 °C and pelleted at 16,000 *g* for 20 min at 4 °C. After washing twice with 75% ethanol, the RNA pellet was resuspended in 15 μl of nuclease-free water. The subsequent steps of vRIC-seq were performed exactly as we previously described^30^, including RNA fragmentation, pCp-biotin selection, and strand-specific library preparation.

### siRNA transfection and RT-qPCR

We synthesized the siRNAs in GenePharma and transfected 10 pmol of each siRNAs into 8 × 10^4^ Vero cells with Lipofectamine RNAiMAX by following the manufacturer’s instructions. The 24-well plates containing the transfected cells were brought to the biosafety level 3 (P3+) facilities for SARS-CoV-2 infection after 24 h. The cells were infected with SARS-CoV-2 (MOI = 0.05) for 1 h at 37 °C in 500 μl Opti-MEM medium. After removing the incubation medium, the infected Vero cells were washed once with Opti-MEM medium and cultured for an additional 24 h in maintenance medium (OPTI-MEM medium containing 1% BSA and 1% penicillin/streptomycin).

For measuring the SARS-CoV-2 levels in the supernatant, 100 μl of virus-containing medium was collected. The viral RNA was extracted with TRIzol LS Reagent (Invitrogen, 10296028) and purified by using Direct-zol™ RNA MiniPrep (ZYMO RESEARCH, R2050) according to the manufacturer’s instructions. The TaqMan RT-PCR assays were performed using TaqMan Fast Virus 1-Step Master Mix (Thermo Fisher Scientific, 4444432). The primers targeting the nucleocapsid (*N*) gene of SARS-CoV-2 and the probe were listed in Supplementary Table 1.

To quantify viral RNA levels inside the cell, we first extracted total RNAs from the infected Vero cells using TRIzol Reagent (Invitrogen, 15596026). For RT-qPCR, 1 μg of total RNA was treated with RQ1 RNase-free DNase (Promega, M6101) and converted into cDNA using MMLV reverse transcriptase (Promega, M1701) with oligo dT (20) primer. qPCR was performed with Hieff qPCR SYBR Green Master Mix (YEASEN, 11203ES08) on a StepOnePlus real-time PCR machine (Applied Biosystems). The primers targeting the *RdRp* gene of SARS-CoV-2 were used for qPCR.

### Processing of vRIC-seq data

vRIC-seq libraries were sequenced by Novogene and the reads were converted into FASTQ format using the bcl2fastq2 software (v2.16). After removing adapters, PCR duplicates, and low-quality sequences, we first aligned the unique vRIC-seq reads to the human 45S pre-rRNA (Refseq accession number NR_046235.3). For all the unmapped reads, we further mapped them to a pan-genome consisting of the *Chlorocebus sabaeus* reference genome (genome assembly version: chlSab2) and the SARS-CoV-2 reference genome (Refseq accession number NC_045512.2) using a STAR software (v020201)^56^. Chimeric reads were identified using the previously described RIC-seq analysis pipeline^30^. Chimeric reads coverage along the genome was visualized by the Circos suite (v0.69-5)^57^.

### The RNA interaction map of SARS-CoV-2

The chimeric reads mapped to the SARS-CoV-2 reference genome were collected and used for generating an RNA interaction map. Of note, we removed spliced reads containing gaps resulted from discontinuous transcriptions^6^. As described previously^30^, we identified the pairwise junction sites in the chimeric reads and used them for building a two-dimensional RNA interaction matrix. This matrix could be converted into .*hic* format and visualized by the Juicebox tool (v1.11.08)^58^. The Knight-Ruiz algorithm^59^ was used to balance this interaction matrix to eliminate sequencing bias along the virus genome.

### Predict local structures of SARS-CoV-2 for validating vRIC-seq data

We used the extended 5′ UTR (1–480 nt) and 3′ UTR (29,546–29,870 nt) sequences to generate candidate secondary structures by the Fold program from RNAstructure software suite (v6.2)^60^. As a general approach, the maximum distance between two paired nucleotides was allowed within 250 nt. The structure that agreed well with the maximized RIC-seq signals between pairwise interacting RNA fragments was selected and visualized by the StructureEditor program (v6.0). The RNAComposer software was applied to deduce the three-way junction’s 3D structure^61^, and the configuration agreed with vRIC-seq data was chosen.

### RNA topological domain in SARS-CoV-2 genome

The SARS-CoV-2 genome was separated into isolated topological domains using our previously published iterative algorithm with minor revisions^30^. Briefly, we iteratively chose the optimal boundary that minimized the inter-domain’s vRIC-seq density as a new candidate domain boundary. To avoid tiny domains resulting from over division, we stopped the iteration once more than 35% of the pairwise 10-nt windows (connection score >0.01) were classified as inter-domain. Lastly, adjacent domains were merged if both did not contain interactions between their 5′ and 3′ boundary.

### MFE analysis of the duplexes revealed by vRIC-seq

To explore whether the pixels with a strong vRIC-seq signal in the RNA interaction map could form long-range duplexes, we first divided the SARS-CoV-2 genome into non-overlapping 10-nt windows. The pairwise 10-nt windows with a connection score >0.01 were used for downstream analyses^30^. Next, we clustered pairwise 10-nt windows adjoining or overlapping at both ends as one interaction. The lowest hybrid free energy was then computed for the possible hybrids formed between these pairwise RNA stretches using the bifold function in the RNAstructure suite (v6.2) with default parameters^60^. Lastly, artificial sequences with the same nucleotide content as real interactions were generated ten times. The lowest hybrid free energy for those shuffled sequences was also calculated.

### 3D structural simulation of SARS-CoV-2 genome

Based on the RNA interaction map, we used the miniMDS program to model the spatial conformation of the SARS-CoV-2 genome using the following parameters^39^: minimds.py -l 10 -m 0.01 -p 0.01. The spatial coordinates reported by miniMDS were smoothed with the LOWESS (locally weighted scatterplot smoothing) algorithm and then visualized^62^. We added a gridded and gray sphere to the modeled 3D structure of the SARS-CoV-2 genome for easy visualization. Pairwise interactions captured by vRIC-seq in local or distal regions were shown as solid or dashed red lines, respectively. Moreover, we also provided a movie illustrating the 3D model of the SARS-CoV-2 genome in virion (Supplementary Video 1).

### SARS-CoV-2 RNA secondary structure modeling

The secondary structure of SARS-CoV-2 genomic RNA was constructed in silico based solely on the vRIC-seq data by an adaptively optimized algorithm we developed in this study. We first split the SARS-CoV-2 genome into shorter segmental domains by maximizing the ratio between intra-domain and inter-domain’s vRIC-seq signals. Notably, the domains smaller than 4 kb will not be further split to avoid the potential loss of long-range duplexes over the domains’ boundaries. Like a previously described approach^15^, we determined the secondary structure for each domain independently. To this end, we systematically screened pairwise 5-nt windows with connection scores higher than 0.03, and the windows adjoining or overlapping at both ends were further clustered as high-confidence interactions. For each interaction spanned region within a domain, we used the Fold program in the RNAstructure software suite (v6.2) to perform structure prediction^60^. The maximum distance between any two paired positions was allowed within 2500 nt. From the structural candidates reported by the Fold program, we selected the one that matched best with vRIC-seq data and forced it as a constraint in the subsequent prediction. Of note, we generated duplexes for short local interactions first and then used them as restraints to perform prediction for long-range interactions spanned regions. Moreover, interactions having stronger vRIC-seq signals were processed with priority. Finally, by restraining duplexes generated in the former stage, we folded each domain’s entire sequence, including regions not covered by the high-confidence interactions. The structure agreed best with vRIC-seq signals were selected. The final secondary structure model of the viral genome RNA in SARS-CoV-2 was visualized by the VARNA program (v3-93)^63^ and the Integrative Genomics Viewer (IGV) visualization tool (v.2.3.92)^64^.

### Evaluate the accuracy of the algorithm

To evaluate the adaptive algorithm’s accuracy, we first used our previously published rRNA+ RIC-seq data in HeLa cells to predict the secondary structure of 28S rRNA^30^. We also used four widely used computational algorithms to predict the secondary structure based on the MFE, including RNAstructure (v6.2)^60^, Mfold (v3.6)^65^, RNAfold^66^, and LinearFold^67^. All parameters were set as default except for the maximum pairing distance within 1600 nt was allowed.

We used two criteria to evaluate a predicted secondary structure’s accuracy: sensitivity and PPV. Sensitivity was defined as the fraction of base pairs that were correctly predicted. PPV was the fraction of base pairs in the predicted structure that were correct. We used a relaxed structure comparison mode. A base pair *i*/*j* was considered correctly predicted if any of the following pairs exist in the reference structure: *i* ± 5/*j* ± 5 and vice versa. The annotated structural model of 28S rRNA was downloaded from the RNAcentral database (https://rnacentral.org/rna/URS000075EC78/9606)^68^.

### Comparison with COMRADES

The COMRADES data generated with SARS-CoV-2 infected Vero E6 cells were downloaded from the Gene Expression Omnibus database under the accession number GSE154662^28^. We analyzed the COMRADES data with the identical pipeline for vRIC-seq data. We used the PPV to compare the interactions captured by vRIC-seq with COMRADES identified duplexes in the host cells. Here, PPV was defined as the percentage of vRIC-seq interactions supported by at least two unique chimeric reads from the COMRADES dataset. The Shannon entropy for each 10-nt genomic window was calculated as previously reported^29^.

### Identification of co-variant base pairs

A total of 429 non-redundant coronavirus genomes were downloaded from the ViPR database^69^ and aligned by the MAFFT program (v7.471)^70^ with default parameters to identify co-variant base pairs in SARS-CoV-2. Based on the multiple sequence alignment results and the SARS-CoV-2 secondary structure derived from vRIC-seq, we employed the cmbuild and cmcalibrate program in the Infernal package (v1.1.3)^71^ to build the covariance model and used the cmserach in the same package to search homologs. Homologs with *E*-value higher than 0.01 were removed, and the alignment of left sequences was subjected to the R-scape program (v1.5.4)^72^ to explore covariance in the SARS-CoV-2. In total, 406 co-variant events were identified (Supplementary Data 3). The alignment file and covariance model are available at https://github.com/caochch/RIC2Structure.

Co-variant base pairs among different SARS-CoV-2 strains were inferred from multiple sequence alignment of 200,621 non-redundant and high-quality SARS-CoV-2 genome sequences downloaded from the Global Initiative for Sharing All Influenza Data (GISAID) database^73^ (date: 7 Jan 2021). A base pair was classified as a co-variant event when both nucleotides were different from the reference SARS-CoV-2 sequence (EPI_ISL_402125) but still base-paired. In total, 98 co-variant events were identified (Supplementary Data 4). Co-variant base pairs were plotted using the R-chie program^74^.

### SNP density and entropy score calculation

SNP annotation for SARS-CoV-2 was downloaded from China National Center for Bioinformation (https://bigd.big.ac.cn/ncov, date: 3 Feb 2021)^75^. In total, this dataset contained 9414 synonymous SNPs. Each nucleotide’s entropy score was calculated according to the multiple sequence alignment of 200,621 SARS-CoV-2 genome sequences. The following formula as previously described was used: *S* = −100 × Sum (*P*_*i*_ × log_2_*P*_*i*_), in which *P*_*i*_ is the frequency of the *i*^th^ allele^69^. Gaps ‘−’ at either end of a sequence in the alignment were removed, and ambiguous residues ‘N’ were excluded from entropy calculation.

### siRNA design

Based on the single-stranded regions revealed by vRIC-seq and the siRNA designing principles described earlier^76^, we randomly selected six siRNAs for experimental validation of SARS-CoV-2 silencing in Vero cells. To explore whether the in-virion SARS-CoV-2 structure could guide siRNA design, we collected 28 siRNAs designed by in silico modeling^36^, 30 siRNAs by the ViennaRNA web server (http://rna.tbi.univie.ac.at/cgi-bin/RNAxs/RNAxs.cgi), as well as all the 331 siRNAs designed from the linear sequence of SARS-CoV-2. Using these collections, we compared the number of single-stranded nucleotides at siRNA-targeted regions in infected cells. We found that the six potent siRNAs target regions also tend to be single-stranded in the host cells.

### Comparison with the in-cell structure

The normalized SHAPE-MaP reactivities on each nucleotide of the SARS-CoV-2 RNA in the infected Vero E6 cells were downloaded from Anna Pyle’s laboratory with the link of https://github.com/pylelab/SARS-CoV-2_SHAPE_MaP_structure^24^. To evaluate the structural dynamics along the SARS-CoV-2 genome, we counted the percentage of shared base pairs within 1 kb sliding windows for every 10 nt along the entire RNA genome. The center of sliding windows moved from the first nucleotide to the 29901st nucleotide. When the center of a sliding window is smaller than 501 or larger than 29401, the 5′ or 3′ end of the sliding window exceeds the boundary of the viral genome. Therefore, we truncated these sliding windows at the endpoint of the viral genome and counted the percentage of shared base pairs within the truncated windows.

### Programmed −1 frameshift assay

The −1 PRF sequence from SARS-CoV-2 was inserted between the coding sequences of renilla luciferase (Rluc) and firefly luciferase (Fluc) to generate a pDual-SARS-CoV-2 plasmid (−1, WT). For the in-frame control reporter pDual-SARS-CoV-2 (0, Ctrl), an additional cytosine was inserted immediately after the silent mutated slippery sequence such that Fluc and Rluc were in the same reading frame (denoted as “0”)^41^. The 33 nt sequence (GGTTATGGCTGTAGTTGTGATCAACTCCGCGAA) that could form a duplex with the Stem 1 region of FSE-PK were deleted by PCR using KOD hot-start high fidelity DNA polymerase (Merck Millipore, 71086) with primers shown in Supplementary Table 1. Frameshifting efficiency was measured by transient transfection of the Dual-Luciferase Reporter plasmids into 293T cells (ATCC, CRL-3216) using Lipofectamine 2000 as previously described^77^. 293T cells were seeded in a 6-well plate at a density of 30%. On the following day, 0.2 μg of each plasmid were individually transfected into cells and allowed for further incubation of 24 h in a 5% CO_2_ incubator. Luciferase activity was measured using a Dual-Luciferase Reporter Assay Kit by following the manufacturer’s instructions (Promega, E1910). We quantified the frameshifting efficiency by normalizing WT and Del values to their in-frame controls as previously described^78^.

### Reporting summary

Further information on research design is available in the Nature Research Reporting Summary linked to this article.

## Supplementary information

Supplementary Information

Supplementary Data1

Supplementary Data 2

Supplementary Data 3

Supplementary Data 4

Supplementary Data 5

Supplementary Movie 1

Description of Additional Supplementary Files

Reporting Summary

## Data Availability

The data supporting the findings of this study are available from the corresponding authors upon reasonable request. vRIC-seq data have been deposited in the Gene Expression Omnibus (GEO) database under accession number GSE155733. Source data are provided with this paper.

## Source data

Source Data

## Code availability

Homemade scripts for reconstructing the secondary structure of SARS-CoV-2 genome and for simulating global configuration could be found at https://github.com/caochch/RIC2Structure.
