# Supplementary material for: The architecture of the SARS-CoV-2 RNA genome inside virion
Source: Nat Commun. 2021 Jun 24;12:3917. doi: 10.1038/s41467-021-22785-x (PMC8225788; doi:10.1038/s41467-021-22785-x)
Supplement: Supplementary file 1 — Supplementary Information [file 41467_2021_22785_MOESM1_ESM.pdf]

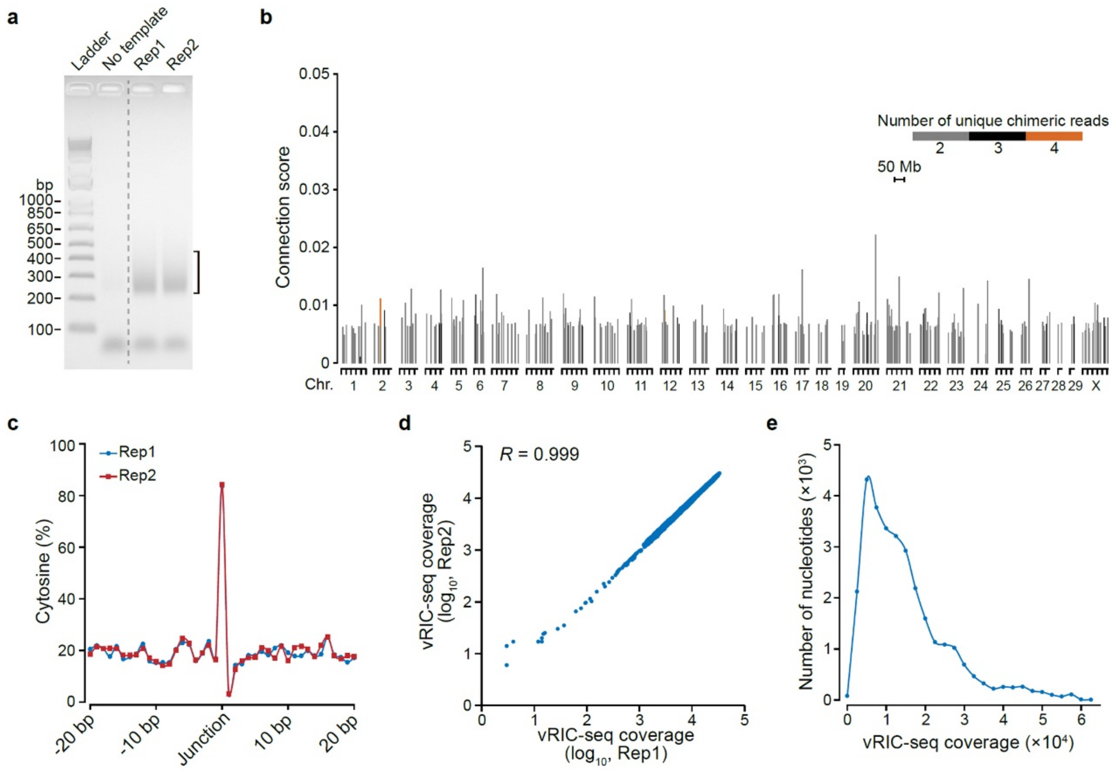

Supplementary Fig. 1 | Characterization of vRIC-seq technology. a, The PCR products were purified from the agarose gel (the bracketed region) for paired-end sequencing. The experiment was independently repeated twice with similar results. Source data are provided as a Source Data file. **b**, The chromosomal location of host RNA fragments ligated to viral RNA. The color scale indicates the number of unique chimeric reads supporting each interaction. Chr represents the chromosome. **c**, The percentages of cytosine around the junction of chimeric reads. **d**, Scatter plot showing the correlation for coverage of chimeric reads along the SARS-CoV-2 genome in two biological replicates (Rep1 and Rep2). *R*, Pearson correlation coefficient. **e**, The coverage of chimeric reads for each nucleotide of the SARS-CoV-2 genome.

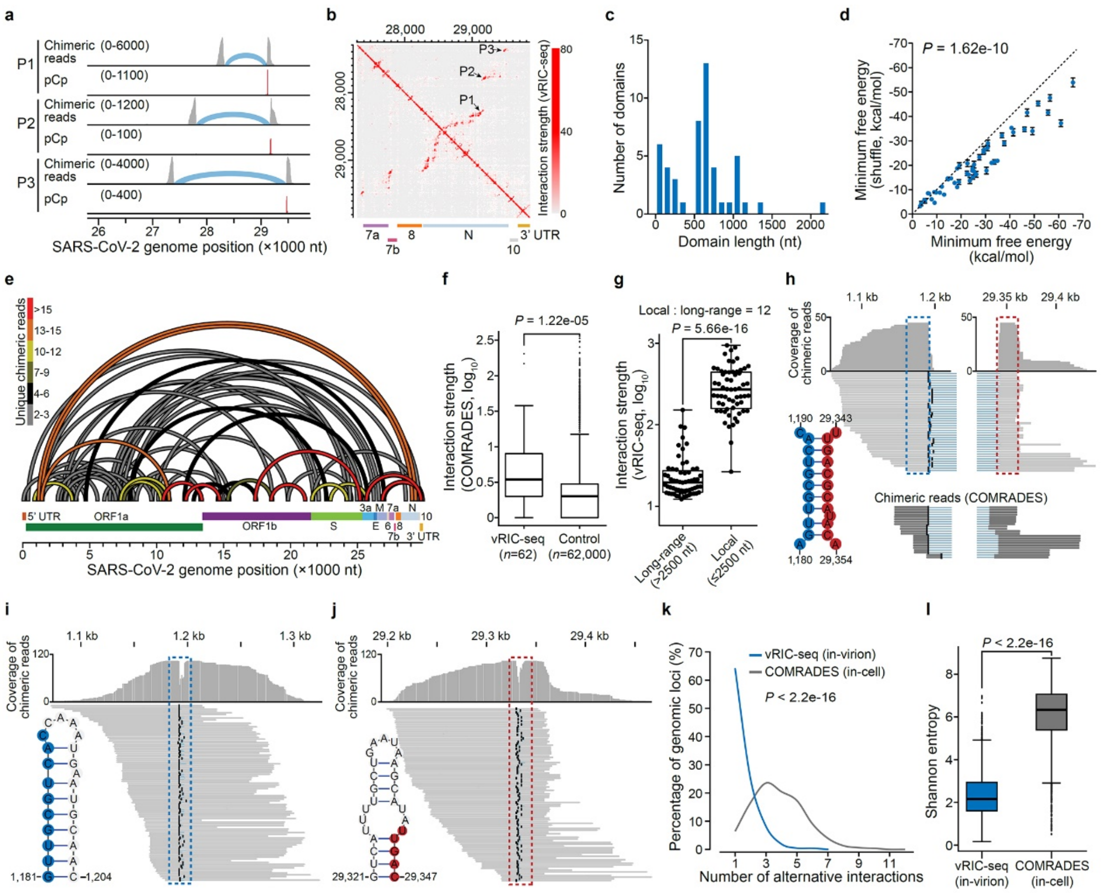

Supplementary Fig. 2 | The features of long-range duplexes. a, Three representative long-rang duplexes contain additional pCp-biotin in the juncture (red lines). **b**, The RNA interaction map shown P1, P2, and P3 duplexes positions. **c**, The size distribution of RNA topological domains. **d**, The minimum free energy of vRIC-seq detected duplexes (n = 52) are significantly lower than the shuffled control. P-value was determined by the two-tailed, paired t-test. Data are mean ± s.e.m. **e**, Schematic diagram showing RNA-RNA interactions spanning more than 2.5 kb along the SARS-CoV-2 genome. The color scale indicates the number of unique chimeric reads supporting each interaction. **f**, vRIC-seq captured long-range interactions (n = 62) showed significantly stronger COMRADES signals than randomly selected size-matched controls (repeated 1000 times, n = 62,000). **g**, RNA fragments spanning more than 2.5 kb showed stronger interaction with its local partners. The median fold of local to long-range interaction is 12. P-value was determined by the two-tailed, paired t-test. n = 62 for long-range and local interactions. **h**, One of the pairwise interactions spanning the longest distance. Chimeric reads from vRIC-seq (grey) and COMRADES (dark grey) are shown at the bottom. The blue line between a chimeric read (grey boxes) represents the gap. A black line is placed at the last base of the left arm to facilitate the visualization of gaps. Bottom left: the predicted hybrids formed between pairwise fragments in the dashed boxes. **i-j**, Chimeric reads support local interactions for 5’ (**i**, blue box) and 3’ (**j**, red box) RNA

fragments shown in (**h**). Bottom: the predicted local duplexes using the RNAstructure program. **k**, COMRADES reveals pervasive alternative RNA-RNA interactions in host cells, while vRIC-seq usually identifies one predominant interaction in the virion. **l**, COMRADES revealed in-cell pairwise interactions showed a higher value of Shannon entropy than in-virion interactions detected by vRIC-seq. The Shannon entropy was calculated for each 10-nt genomic window (n = 2,986). P-values in **f**, **k**, and **l** were determined by the two-tailed, unpaired t-test. For the box plots in **f**, **g**, and **l**, the centre line of the box plot represents the median, the box borders represent the first (Q1) and third (Q3) quartiles, and the whiskers are the most extreme data points within 1.5× the interquartile range (from Q1 to Q3).

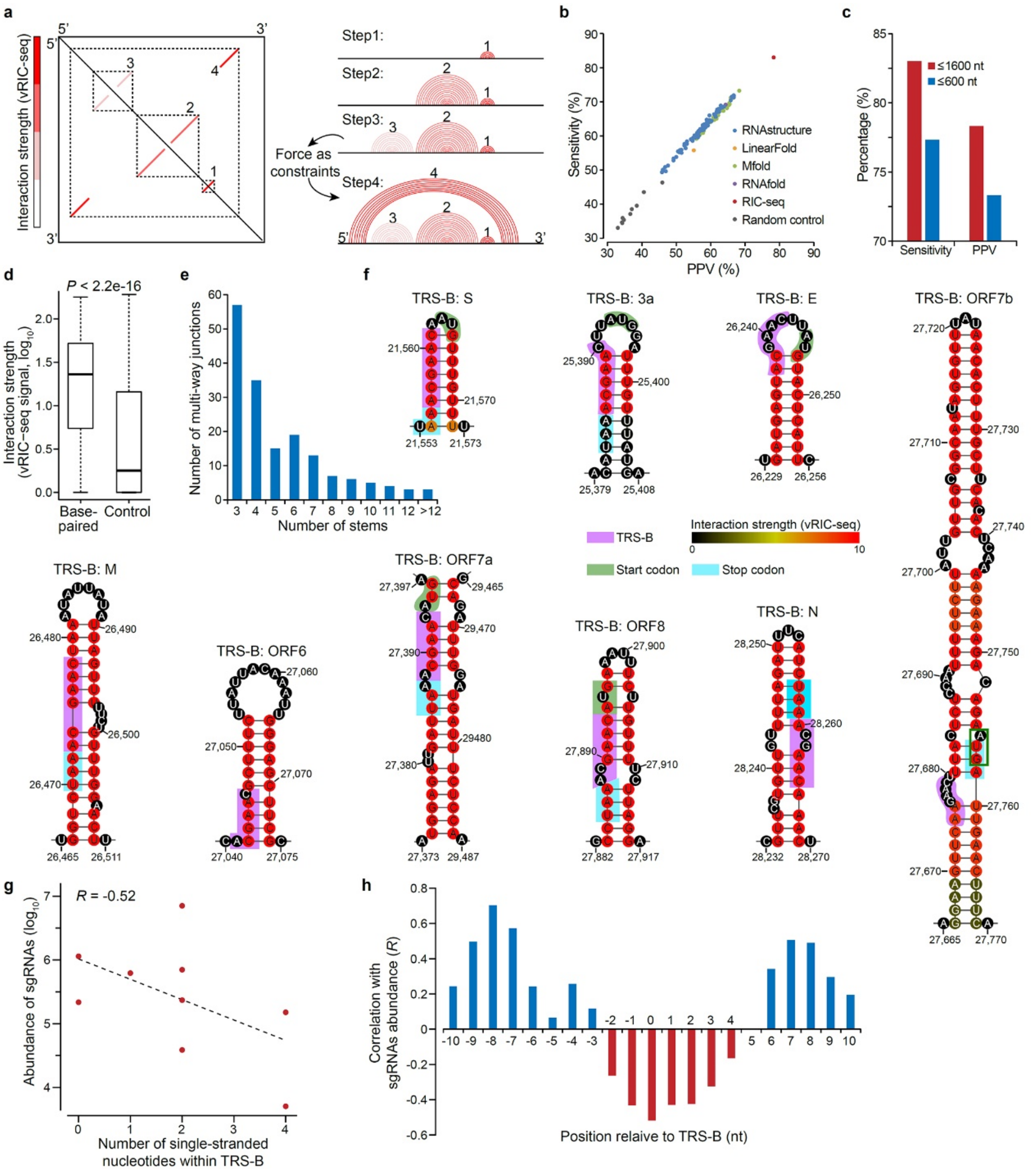

Supplementary Fig. 3 | Performance of the adaptive algorithm. a, The adaptive algorithm was developed to predict RNA secondary structure based on vRIC-seq data. Local duplexes were first predicted and used as a constraint for inferring long-range duplexes. **b**, Comparing the adaptive algorithm's performance and several computational software on predicting the secondary structure of 28S rRNA. PPV stands for positive predictive value. Control was chosen by randomly divide the 28S rRNA into size-matched fragments. **c**, Sensitivity and PPV increased if including the longer duplexes (≤1600 nt, red) for predicting the secondary structure of 28S rRNA by our adaptive strategy. **d**, Pairwise interacting RNA fragments (n = 3,935) showed more vRIC-seq signals than the random controls that have the same spanning distance (n = 22,438). P-value was determined by the two-tailed, unpaired t-test. The centre line of the box plot represents the median, the box borders represent the first (Q1) and third

(Q3) quartiles, and the whiskers are the most extreme data points within 1.5× the interquartile range (from Q1 to Q3). **e**, Summary of the number of multi-way junctions and the corresponding stems in on our secondary structure model. **f**, The secondary structure model of nine TRS-B elements. The core sequence (CS) of each TRS-B, the start codons, and the stop codons are marked by purple, green, and cyan boxes, respectively. **g**, The number of single-stranded nucleotides in the TRS regions showed negative correlations to the sgRNAs abundance. **h**, The number of single-stranded nucleotides surrounding the TRS regions showed positive correlations to the sgRNAs abundance.

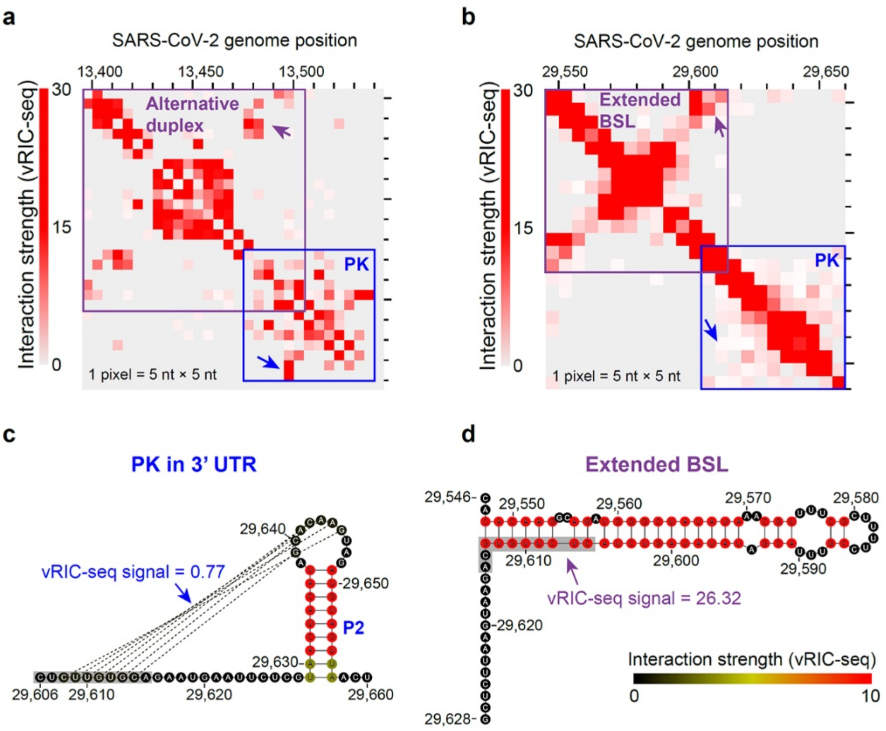

Supplementary Fig. 4 | Alternative conformation for pseudoknots in FSE and 3' UTR. a, The RNA map view of the frame-shifting element (FSE) surrounded regions. Two alternative conformations may mutually present in the virions. Arrowheads are showing two alternative interactions. **b**, The RNA map view of the bulged stem-loop (BSL) element and pseudoknot (PK) in the 3' UTR. The vRIC-seq data preferably supports an extended BSL conformation in the virions (see purple arrowhead). **c**, The 3' UTR pseudoknot structure is barely supported by vRIC-seq data. **d**, An extended BSL duplex is strongly supported by vRIC-seq data.

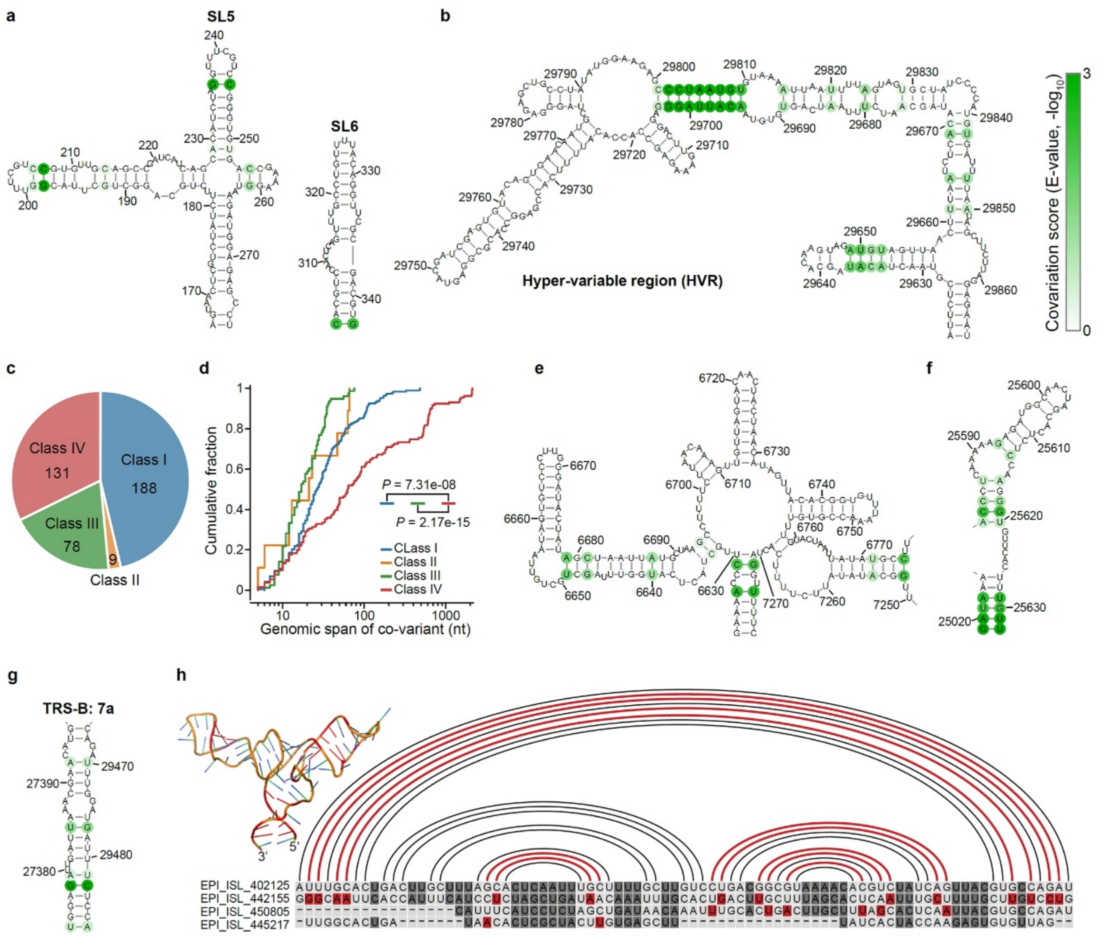

Supplementary Fig. 5 | Co-variant base pairs in SARS-CoV-2. a-b, Base pairs with conserved covariation in the 5' UTR (**a**) and 3' UTR (**b**) are shown in green. Color intensity indicates the covariation score given by the R-scape program. **c**, The in-virion structure revealed more co-variants than the in-cell and in silico predicted structures. **d**, The genomic span distances of two pairwise covariation sites in different categories listed in **c**. Two-sided Kolmogorov-Smirnov test was used to calculate the *P*-value. **e-g**, Co-variant base pairs in three long-range (>600 nt) duplexes. **h**, Co-variant base pairs in a three-way junction (27,552-27,634 nt) in the SARS-CoV-2 genome. Arc lines and nucleotides colored in red indicate co-variant base pairs. RNAComposer modeled the 3D structure of this three-way junction based on vRIC-seq data.

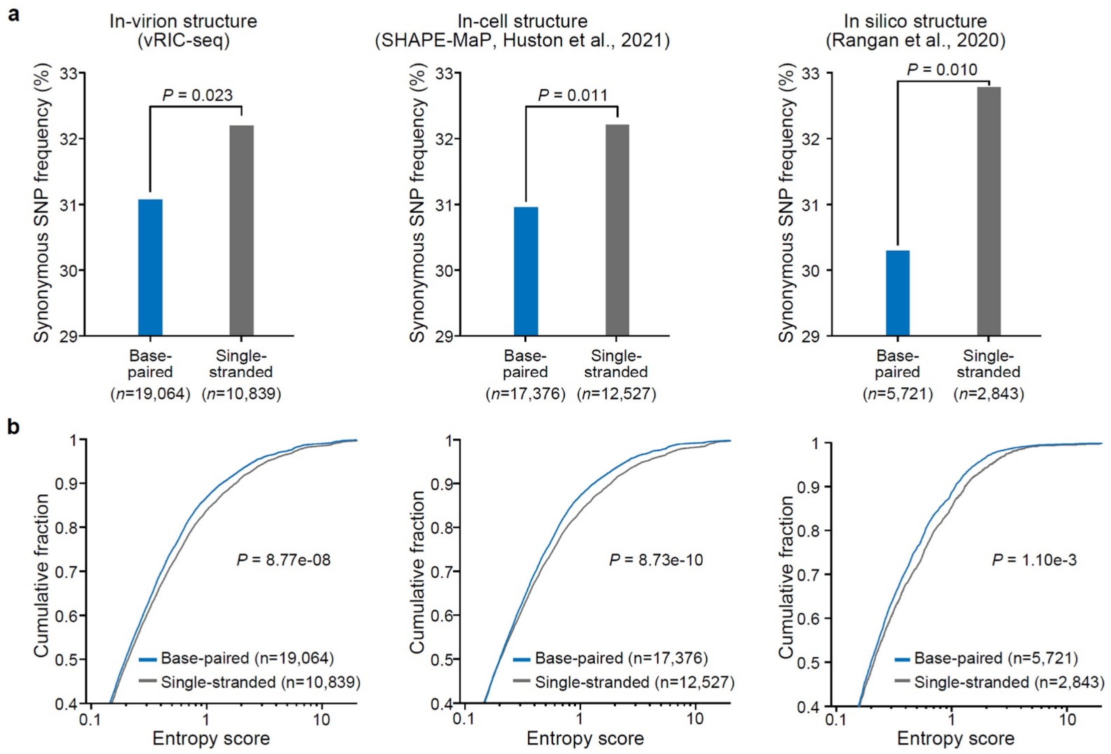

Supplementary Fig. 6 | Base-paired nucleotides are less mutated during evolution. **a**, Synonymous single nucleotide polymorphisms (SNPs) more frequently happen in single-stranded regions than base-paired regions. *P*-value was determined by the one-sided Fisher's exact test. **b**, The entropy scores are significantly lower in the base-paired regions than single-stranded regions. A two-sided Kolmogorov-Smirnov test was used to calculate the *P*-value.

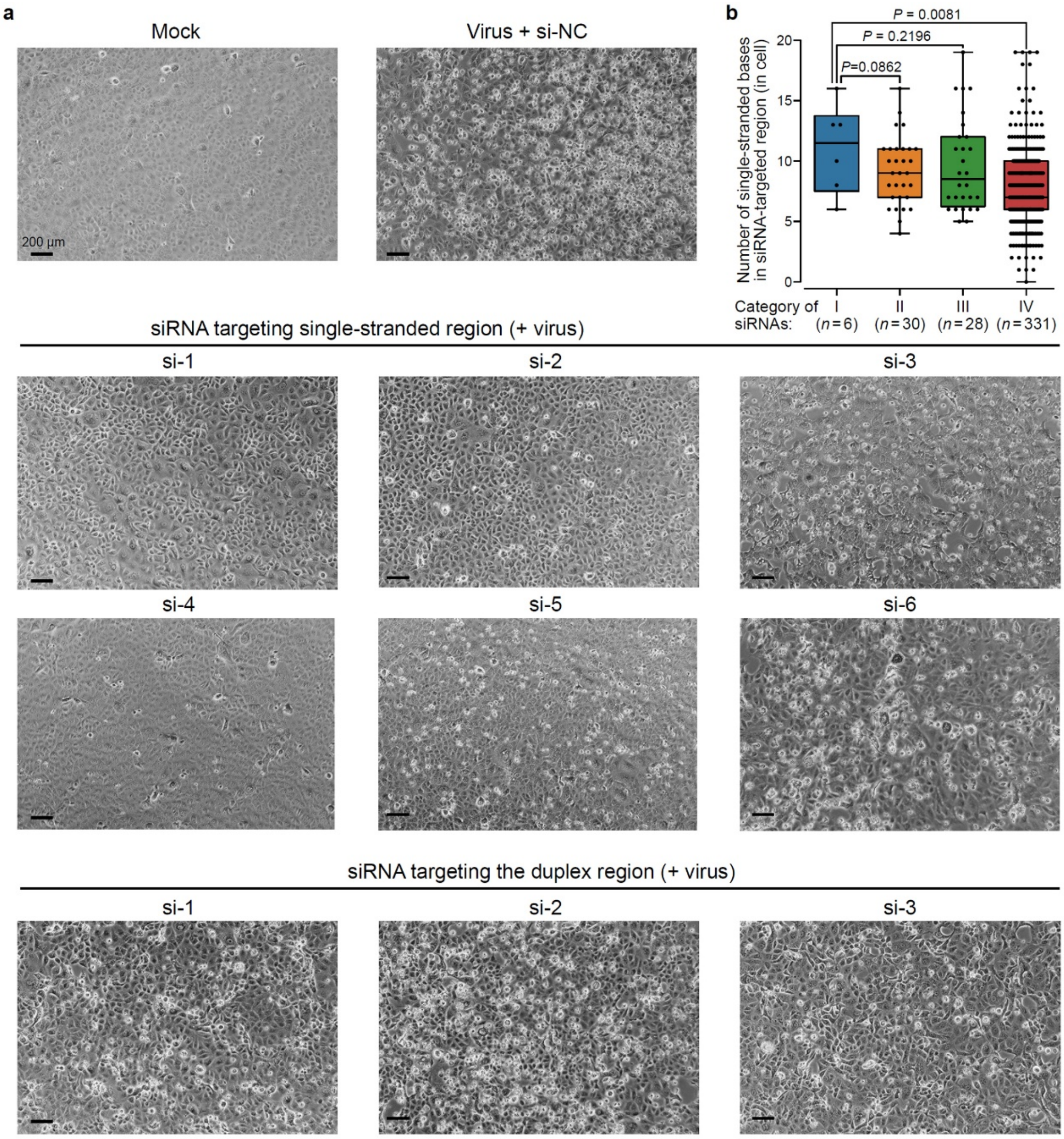

Supplementary Fig. 7 | Cells transfected with single-strand-specific siRNAs are more potent against SARS-CoV-2 infection. a, Vero cells treated by siRNAs targeting single-stranded regions survived better than those with siRNA targeting the duplex regions after the infection of SARS-CoV-2. Scale bar, 200 µm. **b**, The number of single-stranded nucleotides within the siRNAs' target region in cells. Category I: six single-strand-specific siRNAs designed from our in-virion structure model. Category II: siRNAs designed by the ViennaRNA Web Services. Category III: siRNA targeting unstructured regions designed from the in silico model of SARS-CoV-2[36]. Category IV: all possible siRNAs designed from the linear sequence SARS-CoV-2. The centre line of the box plot represents the median, the box borders represent the first (Q1) and third (Q3) quartiles, and the whiskers are the most extreme data points within 1.5× the interquartile range (from Q1 to Q3). *P*-value was determined by the one-tailed, unpaired *t*-test.

Supplementary Table 1. List of siRNAs, primers, and probe used in this study.

siRNA sequences

**siRNA**	**Sequence (5' to 3')**
si-NC	UUCUCCGAACGUGUCACGUTT
single-stranded region si-1	CACACGUCCAACUCAGUUUTT
single-stranded region si-2	GCCCCUUUUCUCUAUCUUUTT
single-stranded region si-3	CCACCACAAACCUCUAUCATT
single-stranded region si-4	GGAGUCAAAUUACAUUACATT
single-stranded region si-5	CACCUGAAGAACAUUUUAUTT
single-stranded region si-6	GAGCCCUAAUGUGUAAAAUTT
duplex region si-1	GGUUGGUGGUAAUUAUAAUTT
duplex region si-2	CUGCAAGAUCAUAAUGAAATT
duplex region si-3	GACUGUGUUAUGUAUGCAUTT

Primer sequences and TaqMan probe used for qPCR

**Primers**	**Sequence (5' to 3')**
pcDNA3.1-dual-1-nCOV delete F	TGTACCGTCTGCGGTATGTGGAAACCCATGCTTCAGTCAGC TGATGCA
pcDNA3.1-dual-1-nCOV delete R	TGCATCAGCTGACTGAAGCATGGGTTTCCACATACCGCAGA CGGTACA
GAPDH-qF	GTCTCCTCTGACTTCAACAGCG
GAPDH-qR	ACCACCCTGTTGCTGTAGCCAA
SARS-CoV-2 *RdRp*-qF	CAAATTCTATGGTGGTTGGCACA
SARS-CoV-2 *RdRp*-qR	GGCATGGCTCTATCACATTTAGG
SARS-CoV-2 *N* gene-qF	AACACAAGCTTTCGGCAGAC
SARS-CoV-2 *N* gene-qR	ACCTGTGTAGGTCAACCACG
SARS-CoV-2 *N* gene probe	/5’-6-FAM/CAGCGCTTCAGCGTTCTTCGGAATGTCGC/3’-BHQ/
