# Supplementary material for: The architecture of the SARS-CoV-2 RNA genome inside virion
Source: Nat Commun. 2021 Jun 24;12:3917. doi: 10.1038/s41467-021-22785-x (PMC8225788; doi:10.1038/s41467-021-22785-x)
Supplement: Supplementary file 8 — Description of Additional Supplementary Files [file 41467_2021_22785_MOESM8_ESM.docx]

Description of additional supplementary information

Title: Supplementary Data 1.

Description: RNA topological domains in the SARS-CoV-2 genome.

Title: Supplementary Data 2.

Description: List of 62 interactions spanning more than 2.5 kb.

Title: Supplementary Data 3.

Description: List of co-variant base pairs among 429 nonredundant coronavirus genomes.

Title: Supplementary Data 4.

Description: List of co-variant base pairs among different SARSCoV-2 strains.

Title: Supplementary Data 5.

Description: List of 130 single-stranded regions in the SARS-CoV-2 genome in virion.

Title: Supplementary Video 1.

Description: The 3D globule configuration of the SARS-CoV-2 genome in virions.
