# Supplementary material for: The architecture of the SARS-CoV-2 RNA genome inside virion
Source: Nat Commun. 2021 Jun 24;12:3917. doi: 10.1038/s41467-021-22785-x (PMC8225788; doi:10.1038/s41467-021-22785-x)
Supplement: Supplementary file 9 — Reporting Summary [file 41467_2021_22785_MOESM9_ESM.pdf]

nature research

Corresponding author(s): Yuanchao Xue

Last updated by author(s): Mar 26, 2021

Reporting Summary

Nature Research wishes to improve the reproducibility of the work that we publish. This form provides structure for consistency and transparency in reporting. For further information on Nature Research policies, see our Editorial Policies and the Editorial Policy Checklist.

Statistics

For all statistical analyses, confirm that the following items are present in the figure legend, table legend, main text, or Methods section.

n/a	Confirmed	
☐	☒	The exact sample size (*n*) for each experimental group/condition, given as a discrete number and unit of measurement
☒	☐	A statement on whether measurements were taken from distinct samples or whether the same sample was measured repeatedly
☐	☒	The statistical test(s) used AND whether they are one- or two-sided<br>*Only common tests should be described solely by name; describe more complex techniques in the Methods section.*
☒	☐	A description of all covariates tested
☒	☐	A description of any assumptions or corrections, such as tests of normality and adjustment for multiple comparisons
☐	☒	A full description of the statistical parameters including central tendency (e.g. means) or other basic estimates (e.g. regression coefficient) AND variation (e.g. standard deviation) or associated estimates of uncertainty (e.g. confidence intervals)
☐	☒	For null hypothesis testing, the test statistic (e.g. *F*, *t*, *r*) with confidence intervals, effect sizes, degrees of freedom and *P* value noted<br>*Give P values as exact values whenever suitable.*
☒	☐	For Bayesian analysis, information on the choice of priors and Markov chain Monte Carlo settings
☒	☐	For hierarchical and complex designs, identification of the appropriate level for tests and full reporting of outcomes
☐	☒	Estimates of effect sizes (e.g. Cohen's *d*, Pearson's *r*), indicating how they were calculated

Our web collection on statistics for biologists contains articles on many of the points above.

Software and code

Policy information about availability of computer code

Data collection	vRIC-seq read sequences were acquired using Illumina instrumentation and software (HiSeq X Ten, bcl2fastq2 Conversion Software v2.16).
Data analysis	All software and code used in this study has been described in published literature (STAR v020201, Circos v0.69-5, Juicebox v1.11.08, Knight-Ruiz algorithm embedded in Juicebox v1.11.08, RNAstructure v6.2, StructureEditor v6.0, RNAComposer http://rnacomposer.cs.put.poznan.pl, miniMDS https://github.com/seqcode/miniMDS, LOWESS algorithm, VARNA v3-93, IGV v2.3.92, Mfold v3.6, RNAfold in ViennRNA v2.4.14, LinearFold https://github.com/LinearFold/LinearFold, MAFFT v7.471, Infernal v1.1.3, R-scape v1.5.4, R-chie https://e-rna.org/r-chie/) or are custom scripts available on GitHub (https://github.com/caochch/RIC2Structure).

For manuscripts utilizing custom algorithms or software that are central to the research but not yet described in published literature, software must be made available to editors and reviewers. We strongly encourage code deposition in a community repository (e.g. GitHub). See the Nature Research guidelines for submitting code & software for further information.

Data

Policy information about availability of data

All manuscripts must include a data availability statement. This statement should provide the following information, where applicable:

- Accession codes, unique identifiers, or web links for publicly available datasets
- A list of figures that have associated raw data
- A description of any restrictions on data availability

vRIC-seq data have been deposited in the Gene Expression Omnibus (GEO) database under accession number GSE155733 (https://www.ncbi.nlm.nih.gov/geo/query/acc.cgi?acc=GSE155733).

Field-specific reporting

Please select the one below that is the best fit for your research. If you are not sure, read the appropriate sections before making your selection.

☒ Life sciences ☐ Behavioural & social sciences ☐ Ecological, evolutionary & environmental sciences

For a reference copy of the document with all sections, see nature.com/documents/nr-reporting-summary-flat.pdf

Life sciences study design

All studies must disclose on these points even when the disclosure is negative.

Sample size	No statistical methods were used to predetermine sample sizes. The sample sizes were determined based on previous experience in the lab or previously published articles. For examples, see Cai et al. (Nature, 2020, 582(7812): 432-437) and Chen et al. (Cell research, 2018, 28(10): 981-995).
Data exclusions	No data was excluded.
Replication	Each experiment was performed independently at least two times. All experiments were highly reproducible.
Randomization	No randomization was required for this study since no comparisons were made between samples/experimental groups. Of note, in vitro cultured cells were treated under the same conditions and randomly assigned to experimental or control groups.
Blinding	Investigators who measured the SARS-CoV-2 copies in the supernatant and the abundance of viral RNA in infected Vero cells were blinded to the siRNA treatment. No randomization was required for other experiments since no comparisons were made between samples/experimental groups.

Reporting for specific materials, systems and methods

We require information from authors about some types of materials, experimental systems and methods used in many studies. Here, indicate whether each material, system or method listed is relevant to your study. If you are not sure if a list item applies to your research, read the appropriate section before selecting a response.

Materials & experimental systems

n/a	Involved in the study	
☒	☐	Antibodies
☐	☒	Eukaryotic cell lines
☒	☐	Palaeontology and archaeology
☒	☐	Animals and other organisms
☒	☐	Human research participants
☒	☐	Clinical data
☒	☐	Dual use research of concern

Methods

n/a	Involved in the study	
☒	☐	ChIP-seq
☒	☐	Flow cytometry
☒	☐	MRI-based neuroimaging

Eukaryotic cell lines

Policy information about cell lines

Cell line source(s)	Vero cell (CCL-81) and 293T cell (CRL-3216) are obtained from ATCC.
Authentication	Cells were not further authenticated.
Mycoplasma contamination	No mycoplasma contamination was detected in Vero or 293T cells.
Commonly misidentified lines (See ICLAC register)	No commonly misidentified cell line was used.
